# Multiplex nucleotide editing by high-fidelity Cas9 variants with improved efficiency in rice

**DOI:** 10.1186/s12870-019-2131-1

**Published:** 2019-11-21

**Authors:** Wen Xu, Wei Song, Yongxing Yang, Ying Wu, Xinxin Lv, Shuang Yuan, Ya Liu, Jinxiao Yang

**Affiliations:** Beijing Key Laboratory of Maize DNA Fingerprinting and Molecular Breeding, Beijing Academy of Agriculture & Forestry Sciences, Shuguang Garden Middle Road No. 9, haidian district, Beijing, China

**Keywords:** CRISPR/Cas9, Base editing, High-fidelity Cas9 variants, tRNA-sgRNA, Off-target effect

## Abstract

**Background:**

Application of the CRISPR/Cas9 system or its derived base editors enables targeted genome modification, thereby providing a programmable tool to exploit gene functions and to improve crop traits.

**Results:**

We report that PmCDA1 is much more efficient than rAPOBEC1 when fused to CRISPR/Cas9 nickase for the conversion of cytosine (C) to thymine (T) in rice. Three high-fidelity SpCas9 variants, eSpCas9(1.1), SpCas9-HF2 and HypaCas9, were engineered to serve with PmCDA1 (pBEs) as C-to-T base editors. These three high-fidelity editors had distinct multiplex-genome editing efficiencies. To substantially improve their base-editing efficiencies, a tandemly arrayed tRNA-modified single guide RNA (sgRNA) architecture was applied. The efficiency of eSpCas9(1.1)-pBE was enhanced up to 25.5-fold with an acceptable off-target effect. Moreover, two- to five-fold improvement was observed for knock-out mutation frequency by these high-fidelity Cas9s under the direction of the tRNA-modified sgRNA architecture.

**Conclusions:**

We have engineered a diverse toolkit for efficient and precise genome engineering in rice, thus making genome editing for plant research and crop improvement more flexible.

## Background

The CRISPR (clustered regularly interspaced short palindromic repeats)/Cas9 system is easily programmed and widely applied for targeted genome editing in human and plant cells [1–3]. The system induces double-strand breaks (DSBs) at a target locus, which triggers homology-directed repair (HDR) or non-homologous end joining (NHEJ) to enable site-directed genome modification [4]. This ability to engineer variation in gene regulatory regions or coding sequences can generate elite trait phenotypes in crops [5–7].

Base editors are powerful tools enabling direct, irreversible conversion of a target DNA base into another and have been widely developed for genome editing [8]. Such precise base-editing technologies largely overcome the inefficiency of homologous-recombination-mediated DNA repair and the challenge of DNA-template delivery into plant cells [9–11]. The first reported cytosine base editor (CBE) was engineered by fusion of rat APOBEC1 (rAPOBEC1) deaminase to the N terminus of SpCas9, which enabled conversion of target C•G base pairs to T•A in the human genome [12]. Soon afterwards, the Kondo group linked the activation-induced cytidine deaminase from sea lamprey (PmCDA1) to the C terminus of SpCas9 nickase to achieve a C-to-T conversion in yeast [13]. These two CBEs were then widely applied in rice and other plants [14–17]. Next, a rAPOBEC1-mediated CBE was developed and optimized using several approaches. Overexpression of a uracil glycosylase inhibitor (UGI) [18] or fusion of two copies of UGI with SpCas9n [19] improved base-editing efficiency and product purity, while modification of nuclear localization signals (NLSs) and codon usage can enhance base-editing efficiency [20, 21]. A new base editor fused with human APOBEC3A cytidine deaminase improved specificity and efficiency in mammalian [22] and plant [23] cells. Generally, rAPOBEC1-related CBEs are more widely applied and exploited than those based on PmCDA1.

Several engineered SpCas9 variants have been developed to minimize off-target effects. eSpCas9(1.1), harboring K848A/K1003A/R1060A mutations, was generated to decrease protein affinity for the non-target DNA strand, thereby decreasing the stability of mismatch-containing helices [24]. SpCas9-HF2, containing N497A/R661A/Q695A/Q926A/D1135E mutations, was designed to decrease the energetics of the SpCas9-single guide RNA (sgRNA) complex to retain robust on-target activity while diminishing the ability to cleave mismatched off-target sites [25]. HypaCas9, possessing N692A/M694A/Q695A/H698A mutations in the REC3 domain, stringently traps the HNH domain at the conformational checkpoint in the presence of mismatches without compromising on-target activity [26]. Moreover, HeFSpCas9s and evoCas9 have been engineered and screened to maintain on-target editing efficiency while exceeding the fidelity of SpCas9 [27, 28]. These refined high-fidelity Cas9 variants may be useful for a broad range of genome-editing applications requiring a high level of specificity.

Effective ways of enhancing the editing activities of high-fidelity Cas9s have also been developed. By linking matched sgRNA to a self-cleaving ribozyme, the editing activity of high-fidelity Cas9 variants can be rescued without sacrificing high specificity [29]. Precise 20-nucleotide guide sequences derived from tRNA–sgRNA precursors also allow these variants to perform robust on-target editing with enhanced specificity [30].

In this study, we compared the performance of PmCDA1 and rAPOBEC1 base-editing systems. In addition, we enhanced the base-editing abilities and random mutational activities of high-fidelity Cas9 variants in rice using tandemly arrayed tRNA-modified sgRNA architecture.

## Results

### PmCDA1 is much more efficient than rAPOBEC1 during base editing in rice

**Fig. 1:**
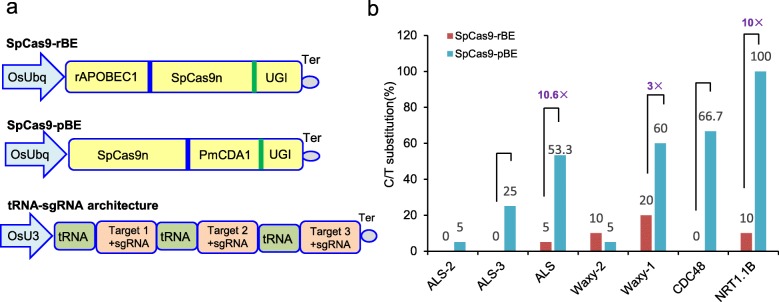
Comparation of the editing efficiency of rBE and pBE. **a** Vector structure used for C-T substitution in both rAPOBEC1 base editing (rBE) and PmCDA1 base editing (pBE) system. tRNA-sgRNA architecture used for multiplex editing was also presented. Three or four targets were assembled to test the editing efficiency. **b** Comparation of C to T substitution efficiency edited by both SpCas9-rBE and SpCas9-pBE system at seven genomic targets guided by wild type sgRNA. For each construction, fifteen transgenic calli were selected independently for Sanger sequencing. Substitution efficiency was defined as the percentage of calli containing C to T conversion at any position in the editing targets

We generated two different CBEs to compare their base-editing efficiencies in rice. SpCas9-rBE was engineered by fusing rAPOBEC1 deaminase to the N terminus of SpCas9n and uracil DNA glycosylase inhibitor (UGI) to the C terminus. PmCDA1 cytidine deaminase and UGI were successively fused to the C terminus of SpCas9n to generate SpCas9-pBE (Fig. 1a). To assess the base-editing efficiencies of these two CBEs, we selected four genes producing classical phenotypes and having the potential to effect crop improvement after mutation of essential amino acids. The acetolactate synthase gene (*OsALS*) confers imazamox herbicide resistance on rice plants when C287 in the coding sequence is replaced by T, which causes an amino acid substitution of Ala96 to Val. This target is henceforth referred to as ALS. Two additional targets, named ALS-2 and ALS-3, were chosen in the same gene. The nitrogen transporter gene (*NRT1.1B*) enhances nitrogen use efficiency in rice when a C-to-T replacement occurs at the 980th cytosine, which causes a Thr327Met mutation. *OsCDC48* regulates senescence and cell death, and a C2347 substitution to T results in a premature stop codon. Two targets named NRT1.1B and CDC48, corresponding to the above two critical sites, were used. The *OsWaxy* gene, an essential enzyme in granule-bound starch biosynthesis, has a profound effect on starch quality and quantity when certain point mutations are induced. Two targets, referred as Waxy-1 and Waxy-2, were chosen for assessment of editing efficiency. Consequently, a total of seven targets were selected from the above four genes. Next, a tandemly arrayed tRNA-sgRNA architecture was designed to produce numerous sgRNAs (Fig. 1a). Nipponbare rice calli were transformed using *Agrobacterium*, and resistant calli were obtained after hygromycin selection. After extraction of genomic DNA from 15 independent transgenic calli, adjacent DNA regions containing the above-mentioned target sites were individually amplified and subjected to Sanger sequencing. The percentage of calli containing a C-to-T conversion in all analyzed calli was defined as the editing efficiency. We obtained an editing efficiency of 0 to 20% using SpCas9-rBE and 5 to 100% with SpCas9-pBE. At most detected targets, the editing efficiency of SpCas9-pBE was 3–10-fold higher than that of SpCas9-rBE (Fig. 1b). Three targets for which editing frequencies have been reported by other laboratories, ALS, CDC48 and NRT1.1B, were edited more efficiently using the SpCas9-pBE system (Additional file 2: Table S1) [14, 15, 17]. The C-to-T editing window located at positions 6 to 8 from the 5′ end of the targets in the SpCas9-rBE system was preferentially situated at positions 1 to 8 in the SpCas9-pBE system (Additional file 1: Figures S1 and S2; Additional file 2: Table S2). The U3 promoter commonly requires an adenine to initiate transcription, but none of the edited targets of the two CBEs began with this nucleotide base (Additional file 2: Table S1). This result indicates that the editing frequency was unaffected by the first base of the targets in the tRNA-sgRNA multiplex system. Collectively, these results demonstrate that SpCas9-pBE is more effective than SpCas9-rBE and has different editing preferences. The SpCas9-pBE construct with tRNA-sgRNA architecture was thus chosen for further study.

### High-fidelity SpCas9 variants and their derived base editors exhibit different efficiencies during multiplex genome editing

**Fig. 2:**
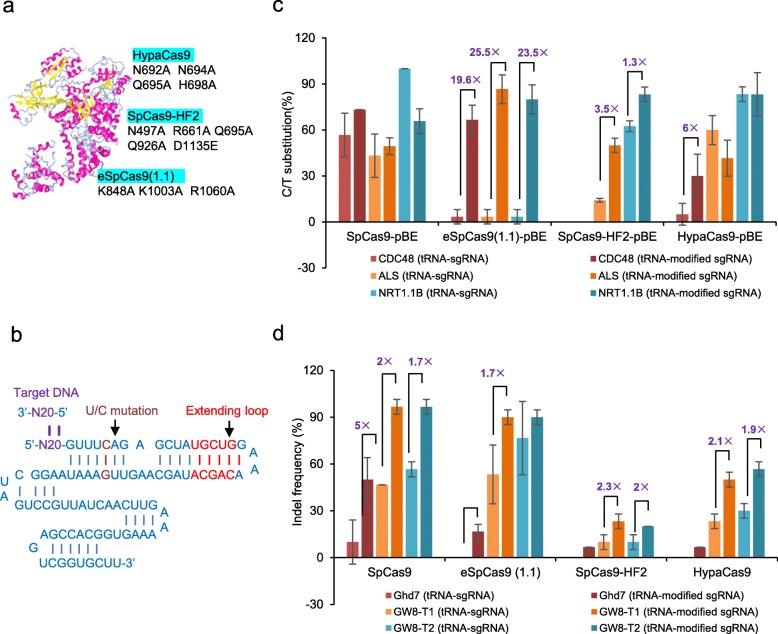
Multiplex nucleotide editing using SpCas9 and sgRNA variants. **a** High-fidelity Cas9 variants with their specific mutation sites used in this study. The crystal structure of SpCas9 protein was download from the structure resource of NCBI (PDB ID: 6O0Y). **b** Schematic representation of modified sgRNA. The polyU mutation was marked with brown and the introduced nucleotides in the extend loop were highlighted in red. N20 with purple indicated targeted DNA. **c** Comparation of C to T editing efficiency of four SpCas9 variants directed by tRNA-sgRNA and tRNA-modified sgRNA in pBE system. Two independent transformations were carried out to repeat the editing efficiency. Substitution efficiency was defined as the percentage of calli containing C to T conversion at any position in the editing targets. Samples with significant increasement were marked out using the black line. **d** Comparation of random mutation efficiency of four SpCas9 variants directed by tRNA-sgRNA and tRNA-modified sgRNA at three targets. Two independent transformations were carried out to repeat the editing efficiency. Indel frequency was defined as the percentage of calli containing deletion or insertion in the editing targets. Samples with significant increasement were marked out using the black line

Next, we engineered three additional high-fidelity base editors (HF-pBEs). Specific amino acid substitutions were introduced to generate the D10A nickases, eSpCas9(1.1), SpCas9-HF2 and HypaCas9 (Fig. 2a). SpCas9n was replaced by these high-fidelity variants to produce eSpCas9(1.1)-pBE, SpCas9-HF2-pBE and HypaCas9-pBE. To check the C-to-T editing efficiency of these three HF-pBEs, we selected the three targets mentioned above from *ALS*, *CDC48* and *NRT1.1B* and used 15 independent transgenic calli. The editing efficiency of HypaCas9-pBE was comparable to that of SpCas9-pBE at ALS (53% vs. 53%) and NRT1.1B (86.7% vs. 100%) sites, while the editing efficiency of SpCas9-HF2-pBE was reduced by at least half (15% vs. 53% at ALS; 65% vs. 100% at NRT1.1B). In contrast, eSpCas9(1.1)-pBE had no base-editing ability at any of the three targets (Additional file 1: Figure S3a). To our surprise, the editing frequency at the CDC48 site was zero for all three HF-pBEs. Consequently, the three HF-pBEs had distinct C-to-T editing efficiencies at the three examined targets in rice.

To test the multiplex knock-out mutation frequency of the high-fidelity SpCas9 variants, we selected one target from the rice genome at Ghd7 and two targets at GW8. SpCas9, the control, exhibited gradable indel activities at the three targets (0, 46.7 and 60% at Ghd7, GW8-T1 and GW8-T2, respectively; Additional file 1: Figure S3b). Compared with the control, the editing efficiency of HypaCas9 was reduced by almost half (0, 26.7 and 26.7% at the three corresponding sites), and SpCas9-HF2 had almost no activity at the targets (0, 6.7 and 6.7%). In contrast, the efficiency of eSpCas9(1.1) was similar to that of SpCas9 (0, 40 and 60%; Additional file 1: Figure S3b). Taken together, these results indicate that the high-fidelity Cas9 variants had different editing abilities at the three examined targets in the random mutagenesis system.

### tRNA-modified sgRNA boosts editing efficiency when combined with the high-fidelity Cas9 variants and HF-pBEs

Because some targets were still resistant to the editing activities of the high-fidelity Cas9s, we set out to improve editing efficiencies at these sites. A modified sgRNA with a fourth T-to-C mutation and a 5-bp extension in the duplex (Fig. 2b) has been reported to be useful for enhancing imaging and knockout efficiency [31–33]. We incorporated this sgRNA into a tRNA–sgRNA system, henceforth referred to as tRNA-modified sgRNA. Compared with the editing efficiency of tRNA-sgRNA, the efficiency of SpCas9-pBE directed by tRNA-modified sgRNA was unchanged at CDC48 and ALS sites but decreased from 100 to 65.7% at the NRT1.1B site (Fig. 2c). Remarkably, tRNA-modified sgRNA greatly enhanced the efficiency of eSpCas9(1.1)-pBE, with a 19.6- to 25.5-fold increase detected at all three sites. As a consequence, eSpCas9(1.1)-pBE was as powerful as SpCas9-pBE at the three targets. In regard to SpCas9-HF2-pBE, guidance by tRNA-modified sgRNA increased the substitution frequency at ALS and NRT1.1B sites by 3.5- and 1.3-fold, respectively, whereas no effect was observed at CDC48. The editing efficiency of HypaCas9-pBE under the direction of tRNA-modified sgRNA was significantly enhanced at the CDC48 site, from 5 to 30%, while that at the other two sites was unchanged (Fig. 2c). Substitution preferences at the three examined targets were unaffected by tRNA-modified sgRNA in all HF-pBEs (Additional file 2: Table S3), but a high proportion of random mutational events occurred at the NRT1.1B site with all base editors (Additional file 2: Table S4 and Additional file 1: Figures S4 and S5).

We also analyzed the effect of tRNA-modified sgRNA in the random mutagenesis system. The editing efficiencies of all three high-fidelity Cas9 variants were enhanced two- to three-fold at the different targets. Consequently, the activity of eSpCas9(1.1) was comparable to that of SpCas9 (Fig. 2d). In particular, tRNA-modified sgRNA improved the random mutational efficiency of SpCas9 but had no effect on SpCas9-pBE. Overall, these results demonstrate that tRNA-modified sgRNA greatly facilitated the C-to-T base-editing ability or random mutational efficiency of high-fidelity Cas9s, especially that of eSpCas9(1.1), at the three examined targets.

### Specificities of Cas9 variants in combination with tRNA-modified sgRNA

**Fig. 3:**
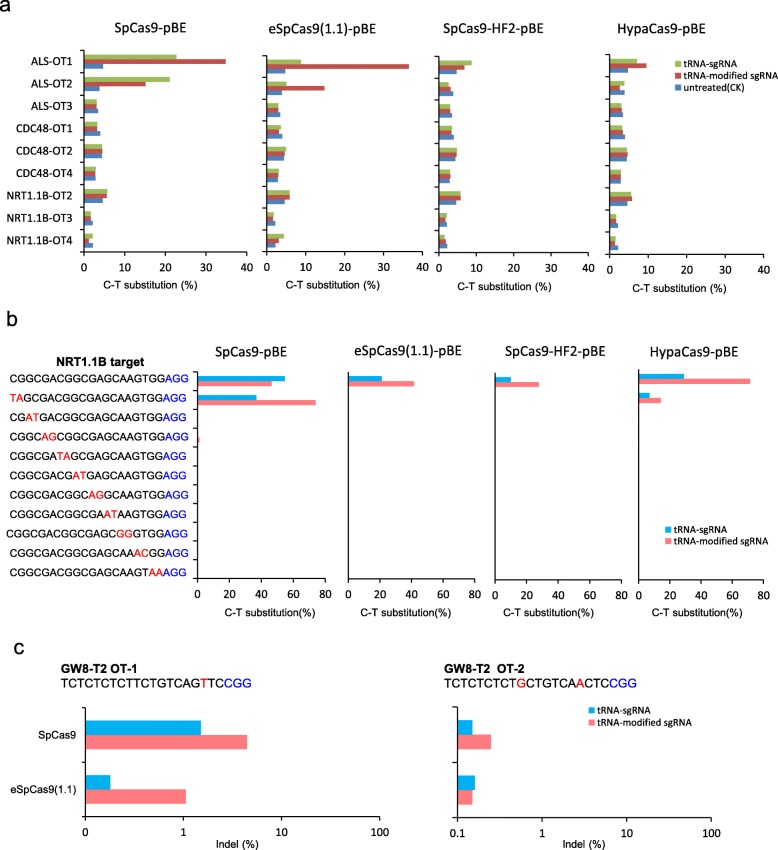
Specificities of high-fidelity Cas9 variants. **a** Off-target activity detected at three potential off-target sites of ALS, CDC48 and NRT1.1B in the genomics in pBE system. The off-target efficiency was detected using deep sequencing described in method. **b** Off-target effects of four Cas9 variants detected with guide sequences containing pairs of mismatches at successive positions combined with tRNA-sgRNA and tRNA-modified sgRNA in pBE system. The mismatch sites were highlighted in red with the PAM highlighted in blue. **c** Off-target activity of SpCas9 and eSpCas9(1.1) detected on two potential off-target sites of GW8-T2 in the genomics using deep sequencing during random mutation

There is a potential risk of stimulation of off-target activities by tRNA-modified sgRNA; therefore, we examined three predicted off-targets of each base-editing site. The DNA of eight independent transgenic calli were mixed, and the off-target sequences were amplified for deep sequencing. Calli from wild-type Nipponbare were used as a negative control. Two potential off-targets of ALS (ALS-OT1 and ALS-OT2) harbored only one base that was different from the ALS on-target sequence, while the other off-targets differed by at least two bases from their on-target sequence. When SpCas9-pBE was guided by tRNA-modified sgRNA, the off-target frequency of ALS-OT1 increased by 1.5 fold (from 22.7 to 34.8%), while a 28% reduction (from 21 to 15%) was observed at the ALS-OT2 site compared with the performance of tRNA–sgRNA. Unexpectedly, tRNA-modified sgRNA sharply increased the off-target activity of eSpCas9(1.1)-pBE at both ALS-OT1 and ALS-OT2 sites (from 1.83 to 11.61% and 1 to 28.73%, respectively) (Fig. 3a; Additional file 2: Table S5). The specificities of SpCas9-HF2-pBE and HypaCas9-pBE were, however, largely unaffected by tRNA-modified sgRNA. In addition, no obvious off-target effect was observed with any base editors at the detected targets of CDC48 and NRT1.1B (Fig. 3a). According to these results, tRNA-modified sgRNA increased the off-target effect of SpCas9-pBE and eSpCas9(1.1)-pBE at predicted off-targets differing by one base pair from the on-target. Nevertheless, the fidelity of SpCas9-HF2-pBE and HypaCas9-pBE remained high at all analyzed off-targets even when guided by tRNA-modified sgRNA.

To further confirm the editing specificity of the high-fidelity variants of the base-editing tools, we modified the NRT1.1B target, which had the highest substitution efficiency, to incorporate different paired mismatches (Fig. 3b). Rice calli were transformed using *Agrobacterium*, and resistant calli were obtained after hygromycin selection. DNA of 8 to 10 independent calli were mixed, and the NRT1.1B target sequences were amplified for deep sequencing. Obvious off-target effects were observed with SpCas9-pBE when two different bases were present at the 5′ terminus of the target, whereas lowered or no off-target effects were observed at the same targets with eSpCas9(1.1)-pBE, SpCas9-HF2-pBE or HypaCas9-pBE (Fig. 3b). This result indicates that the three high-fidelity base editors still had a higher specificity than SpCas9-pBE when the off- and on-targets differed by two bases.

Because the knock-out activities of SpCas9-HF2 and HypaCas9 under the direction of either tRNA-sgRNA or tRNA-modified sgRNA were lower compared with wild-type SpCas9, we chose the best eSpCas9(1.1) to analyze off-target frequencies when guided by either tRNA-sgRNA or tRNA-modified sgRNA. Eight predicted off-target sites of GW8-T1 and GW8-T2 were detected by deep sequencing as described above (Additional file 2: Table S6). No obvious off-target activity was detected at the four off-sites of GW8-T1 (data not shown). At the two off-sites of GW8-T2 (referred to as GW8-T2-OT1 and GW8-T2-OT2), tRNA-modified sgRNA increased the off-target efficiency of both SpCas9-pBE and eSpCas9(1.1)-pBE. Nevertheless, the specificity of eSpCas9(1.1)-pBE was still higher than that of SpCas9-pBE without compromising the high random mutational efficiency (Fig. 3c).

## Discussion

In plant and animal cells, base editing is more efficient than conventional nuclease-mediated HDR-dependent genome editing [8]. The deamination activity of three different cytidine deaminase enzymes, rAPOBEC1, PmCDA1 and hAPOBEC3A, fused with SpCas9 variants has been examined in eukaryotes [12, 13, 22]. In plants, the rAPOBEC1-based CBE has been widely applied [14–16]. Here, we found that the base-editing activity of the PmCDA1 system is much more efficient in rice compared with that of rAPOBEC1, which is contrary to findings in human cells [19]. The nuclease components of SpCas9-rBE and SpCas9-pBE were all optimized for rice by GenScript (Nanjing) and were individually driven by the same OsUbq promoter; therefore, the expression levels of the two editors should not have differed significantly from one another. In addition, data were collected from many independent cells under the same transformation conditions to avoid the influence of T-DNA insertion sites. Consequently, our finding that PmCDA1 is more efficient is convincing. According to two previous reports, improvement of editor protein expression through modification of NLSs and codon usage can enhance the editing efficiencies of the rAPOBEC1 system in human cells. To improve the editing efficiency of SpCas9-rBE, future measurement of nuclease expression levels would therefore be helpful [20, 21]. The effective deamination window used by SpCas9-pBE extended from positions 1 to 8 beginning from the 5′ end of the protospacer, while positions 6 to 8 in the examined targets were preferred by SpCas9-rBE. In a recent study, a hAPOBEC3A-based CBE had a higher editing efficiency than rAPOBEC1 and used a 17-nucleotide editing window at examined sites in plants [23]. These additional features may be helpful and optional for inducing multiple point mutations in plant genomes. However, because the wider activity window may lessen the precision of single substitutions, more extensive mutations of PmCDA1 cytidine deaminase need to be created and tested in future studies.

Modified sgRNA can increase efficiencies for the enhancement of both imaging and gene regulation in human cells [33]. When combined with wild-type SpCas9 or a SpCas9-VQR variant, modified sgRNA also improves knock-out activity in rice and mammalian cells [31, 32]. Gao et al. have recently reported that a modified sgRNA leads to higher editing efficiency compared with either native sgRNA or tRNA-native sgRNA in an adenine base editor (ABE) in plants [34]. Although SpCas9n is used in the ABE system, we found that tRNA-modified sgRNA architecture enhanced SpCas9n-mediated cytidine base editing only slightly in just one of three examined targets. These conflicting results indicate that different deaminases fused with SpCas9n may affect the interaction between SpCas9n and sgRNA or modified sgRNA, thus leading to divergent effects on base editors. Another interesting phenomenon is that the same high-fidelity Cas9s have different base editing and random mutagenetic performances. For example, eSpCas9(1.1) had a mutational efficiency in the random mutagenesis system that was roughly the same as that of SpCas9, whereas its editing ability in the base-editing system was relatively low. This inconsistency is because of the divergent contributions of tRNA-modified sgRNA between different systems (Fig. 2c and d). Given that only a few targets were examined, additional sites should be tested to further confirm the effect of tRNA-modified sgRNA on SpCas9-pBE and HF-pBE.

Although high-fidelity SpCas9 variants have been engineered to achieve high genome-wide specificity in human cells without compromising on-target activity, the on-target activities of knock-out mutations are sometimes considerably lower in plants [30]. Here, we evaluated the C-to-T conversion efficiency of three high-fidelity Cas9 variants fused with PmCDA1 and found that base-editing abilities were decreased compared with those of the wild-type base editor. However, a tRNA-modified sgRNA dramatically increased the base-editing activity of eSpCas9(1.1)-pBE and SpCas9-HF2-pBE, from 1.3- to 25.5-fold. This differential enhancement indicates that the modified sgRNA acted independently with different Cas9s in each editing system, a finding requiring further confirmation with more targets. Inspired by this significant enhancement of eSpCas9(1.1) by tRNA-modified sgRNA, a future study should combine artificially developed sgRNAs with different Cas9 variants to boost editing efficiency.

Finally, we examined the off-target effects of SpCas9 variants in the base-editing system. tRNA-modified sgRNA substantially improved the on-target editing efficiency of eSpCas9(1.1)-pBE but had limited effect on SpCas9-pBE. Off-target efficiency, however, was increased for both SpCas9-pBE and eSpCas9(1.1)-pBE directed by tRNA-modified sgRNA at predicted off-targets that differed by only one base pair from the target site. In contrast, the three high-fidelity base editors still operated with higher specificity than SpCas9-pBE when two base differences existed between off- and on-targets. Only limited results were obtained by deep sequencing. Consequently, an off-site mutation analysis on a more global scale using whole genome sequencing is needed to further understand the off-target effects of the different SpCas9 variants.

## Conclusions

In this study, we compared the base-editing efficiency of PmCDA1 and rAPOCEB1 in CBEs in rice. According to our results, the PmCDA1 system is more advantageous than the rAPOBEC1-based system. We evaluated the base-editing and off-target efficiency of three high-fidelity SpCas9 variants directed by tRNA-modified sgRNA. The tandemly arrayed tRNA-modified sgRNA architecture substantially improved the base-editing efficiency of these high-fidelity base editors by up to 25.5-fold. For targets having potential off-sites differing by at least two base pairs, the combination of tRNA-modified sgRNA and eSpCas9(1.1)-pBE constitutes a superior tool with a higher editing efficiency for C-to-T conversion. When higher specificity is needed during editing, modified sgRNA can be combined with SpCas9-HF2-pBE or HypaCas9-pBE. To achieve random mutagenesis with higher editing efficiencies and lower off-target effects in plants, eSpCas9(1.1) guided by tRNA-modified sgRNA may be more suitable than tRNA-sgRNA-guided SpCas9. These diverse editing tools enhance the flexibility of precise base editing and random mutagenesis for plant research and crop improvement.

## Methods

### Plasmid construction

All vectors were constructed with a modified pCambia2300 backbone, in which the BsaI site was eliminated and the kanamycin-resistance gene was replaced with a spectinomycin-resistance gene. KpnI and SbfI were used to connect T-DNA cassettes to the backbone. Most components not amplifiable from the rice genome were codon-optimized and synthesized by GenScript (Nanjing, China). Four additional fragments, detailed below, were generated, fused and inserted into the modified backbone to produce the SpCas9-pBE basic construct. The OsU3 promoter flanked by KpnI and BamHI was synthesized together with tRNA-sgRNA architecture and the OsU3 terminator, the total of which was digested with KpnI and HindIII to yield fragment 1. To generate fragment 2, the OsUbq promoter (LOC02g06640) flanked by HindIII and SnaBI was amplified from the Nipponbare genome. SpCas9 nickase fused with PmCDA1 and UGI and appended to a 35S terminator was synthesized and digested with SnaBI and AvrII (fragment 3). Finally, the ZmUbi1 promoter was amplified from the B73 genome, and a hygromycin-resistance gene and its terminator were amplified from pCambia2300. These two elements were fused using an Infusion kit (Takara; cat. no. 639686) and digested with AvrII and SbfI to give fragment 4. The four fragments were ligated together with digested backbone (KpnI and SbfI) using T4 DNA ligase (NEB; cat. no. M0202 L) to generate the SpCas9-pBE basic construct (Additional file 1: Figure S6a). To produce the SpCas9-rBE basic construct, three fragments (5–7) were prepared. rAPOBEC1 was synthesized and digested with SnaBI and BsaI to generate fragment 5. Fragment 6 was formed by amplifying SpCas9n flanked by two BasI sites using SpCas9-pBE plasmid as a template. To create fragment 7, UGI and the 35S terminator were amplified using the SpCas9-pBE plasmid as a template and then digested with BasI and AvrII. BsaI, a type-II endonuclease with different recognition and cutting sites, was used to link fragments 5, 6 and 7 without additional sequences. The SpCas9-pBE basic construct digested with SnaBI and AvrII was used as a backbone for ligation with fragments 5–7 to generate the SpCas9-rBE basic construct (Additional file 1: Figure S6b). All PCR and enzyme digestion products were purified using an AxyPrep DNA Gel Extraction kit (Axygen; AP-GX-250G). Multiplex editing targets were cloned before the sgRNA using BsaI as described previously [35]. Point mutations were induced using the Fast MultiSite Mutagenesis system (TransGen Biotech, Beijing, China) to generate eSpCas9(1.1), SpCas9-HF2 and HypaCas9 nickase variants (Fig. 1c). All essential sequences are listed in Additional file 1: Figure S7. For the coupling of constructs with paired mismatches to NRT1.1B, 20-bp target sequences were synthesized and annealed in a PCR thermal cycler in reactions consisting of 1 μl forward oligo (100 μM), 1 μl reverse oligo (100 μM), 1 μl 10× T4 DNA ligase buffer and 7 μl H_2_O. The annealing protocol consisted of 37 °C for 60 min and 95 °C for 10 min, followed by cooling to 25 °C at a rate of 0.1 °C s^− 1^. The annealed oligo adaptors were inserted into the BsaI-digested backbone, and the final plasmid was confirmed by Sanger sequencing. All primers used in this study are listed in Additional file 2: Table S7. Essential sequences can be found in Additional file 1: Figure S7.

### Rice transformation

Japonica seeds (Nipponbare) were originally acquired from Professor Fengyi Hu, College of Agriculture, Yunnan University, China. Induced calli were used for *Agrobacterium* mediated transformation using LBA4404, as previously described [36]. Incubated and recovered calli were selected on 50 mg/ml hygromycin for 4 weeks to obtain resistant calli.

### Mutation frequency detection

Genomic DNA of resistant calli was extracted using the DNA quick Plant System (TIANGEN BIOTECH, Beijing, China). Target sequences were amplified using specific primers and the PCR products were sent for sequencing to detect the mutation frequency of each construction.

### Off-target detection

For first generation sequencing, fragments with potential off-target sites were amplified from rice genomic DNA and purified using the EasyPure PCR Purification Kit (TransGen Biotech, Beijing, China). The products were ligated into pEASY-B (TransGen Biotech, Beijing, China) and 100 clones were selected for Sanger sequencing to determine the off-target efficiency. For deep sequencing, the on-target and off-target regions were amplified using specific primers. Barcodes were then added for next generation sequencing library construction. Pooled PCR amplicons were sequenced using the Illumina NextSeq platform. Indel mutations occurring during base editing were excluded from the substitution efficiency.

### Sequence analysis

For deep sequencing, raw data was filtered by Filter_FQ to remove unqualified reads, including sequences with an N base ratio greater than 10% or with more than 50% low quantity bases. The filtered data was aligned to the IRGSP 1.0 genome with BWA (http://bio-bwa.sourceforge.net/) using default parameters. Samtools mpileup (http://samtools.sourceforge.net/) was employed for SNP identification [37]. An in-house script was used to calculate the mutation frequency and mutation type for each locus. For Sanger sequencing, results were aligned and analyzed using DSDecode [38]. C-to-T mutation frequency in calli was defined as the percentage of mutants with any target C-to-T substitution in all transgenic samples. Indel frequency was the percentage of mutants with any indels among the C-to-T mutants.

## Supplementary information

**Additional file 1:Figure S1.** Base editing results of SpCas9-pBE system. Genes, target sequences and sequencing results of six editing targets were showed in SpCas9-pBE system. PAM sequence was highlighted in green, the C to T conversion bases were highlighted in blue. Red arrow indicated the mutation peak. The results were from 15 positive calli. **Figure S2**. Base editing results of SpCas9-rBE system.Genes, target sequences and sequencing results of four editing targets were showed in SpCas9-rBE system. PAM sequence was highlighted in green, the C to T conversion bases were highlighted in blue. Red arrow indicated the mutation peak. The results were from 15 positive calli. **Figure S3**. On-target activities of high fidelity SpCas9 variants guided by wild-type sgRNA.a C-T substitution frequency of SpCas9-pBE and high fidelity Cas9 pBEs at three genomic targets. b Random mutation frequency of SpCas9 and three variants at three genomic targets. All the frequencies were calculated among 15 positive calli. **Figure S4**. C-T substitution and random mutation occurred at NRT1.1B site in pBE system. Three samples with random mutations were selected from the calli of SpCas9-pBE and eSpCas9(1.1)-pBE complexed with the modified sgRNA each to detect the actual mutation types. PCR product were cloned to pEASY-B vetor and 27 positive clones were sent for sequencing. Arrows indicated the  substitution base. **Figure S5**. Random mutation frequency at NRT1.1B site in pBE system. Three samples with random mutations were selected from the calli of SpCas9-pBE and eSpCas9(1.1)-pBE complexed with the modified sgRNA each. Proportions of C-T substitution (SNP), random mutation (Indel), and substitution mixed with random mutation (SNP + Indel) were shown from 27 positive B-vector cloing  above. **Figure S6**. Schematic diagram for the constructions of base editors. Fragments, backbones and related restriction enzymes used in the construction of SpCas9-pBE (a) and SpCas9-rBE (b) base editors. **Figure S7**. Essential sequences used in this study.

**Additional file 2 **: **Table S1.** Comparation of base editing efficiency in our study with reported results. **Table S2.** Base editing preference of SpCas9-rBE and SpCas9-pBE in rice. **Table S3.** Precise base editing preferences and frequencies in all SpCas9 base editors on OsCDC48, OsALS and OsNRT1.1B sites. **Table S4.** The frequency of random mutation occurred during precise base editing on OsCDC48, OsALS and OsNRT1.1B sites in all SpCas9 base editors. **Table S5.** Potential off-target sites in rice genomics for base editing targets. **Table S6.** Potential off-target sites in rice genomics for random mutation targets. **Table S7.** Primers used in this study.

## Data Availability

Deep sequencing data are available under BioProject ID: SRP147977. The website is: https://www.ncbi.nlm.nih.gov/sra/?term=SRP147977. Data has already been released to the public.

## Abbreviations

CBE: Cytidine base editor
HF pBEs: High-fidelity base editors
OT: Off-target sites
pBE: Base editing system mediated by PmCDA1 cytidine deaminase
rBE: Base editing system mediated by rAPOBEC1 cytidine deaminase
sgRNAs: Single guide RNAs; SpCas9n: SpCas9 nickase
