# Supplementary material for: Multiplex nucleotide editing by high-fidelity Cas9 variants with improved efficiency in rice
Source: BMC Plant Biol. 2019 Nov 21;19:511. doi: 10.1186/s12870-019-2131-1 (PMC6873407; doi:10.1186/s12870-019-2131-1)
Supplement: Supplementary file 1 — Additional file 1:Figure S1. Base editing results of SpCas9-pBE system. Genes, target sequences and sequencing results of six editing targets were showed in SpCas9-pBE system. PAM sequence was highlighted in green, the C to T conversion bases were highlighted in blue. Red arrow indicated the mutation peak. The results were from 15 positive calli. Figure S2. Base editing results of SpCas9-rBE system.Genes, target sequences and sequencing results of four editing targets were showed in SpCas9-rBE system. PAM sequence was highlighted in green, the C to T conversion bases were highlighted in blue. Red arrow indicated the mutation peak. The results were from 15 positive calli. Figure S3. On-target activities of high fidelity SpCas9 variants guided by wild-type sgRNA.a C-T substitution frequency of SpCas9-pBE and high fidelity Cas9 pBEs at three genomic targets. b Random mutation frequency of SpCas9 and three variants at three genomic targets. All the frequencies were calculated among 15 positive calli. Figure S4. C-T substitution and random mutation occurred at NRT1.1B site in pBE system. Three samples with random mutations were selected from the calli of SpCas9-pBE and eSpCas9(1.1)-pBE complexed with the modified sgRNA each to detect the actual mutation types. PCR product were cloned to pEASY-B vetor and 27 positive clones were sent for sequencing. Arrows indicated the substitution base. Figure S5. Random mutation frequency at NRT1.1B site in pBE system. Three samples with random mutations were selected from the calli of SpCas9-pBE and eSpCas9(1.1)-pBE complexed with the modified sgRNA each. Proportions of C-T substitution (SNP), random mutation (Indel), and substitution mixed with random mutation (SNP + Indel) were shown from 27 positive B-vector cloing above. Figure S6. Schematic diagram for the constructions of base editors. Fragments, backbones and related restriction enzymes used in the construction of SpCas9-pBE (a) and SpCas9-rBE (b) base editors. Figure S7. Essen [file 12870_2019_2131_MOESM1_ESM.pptx]

## Slide 1
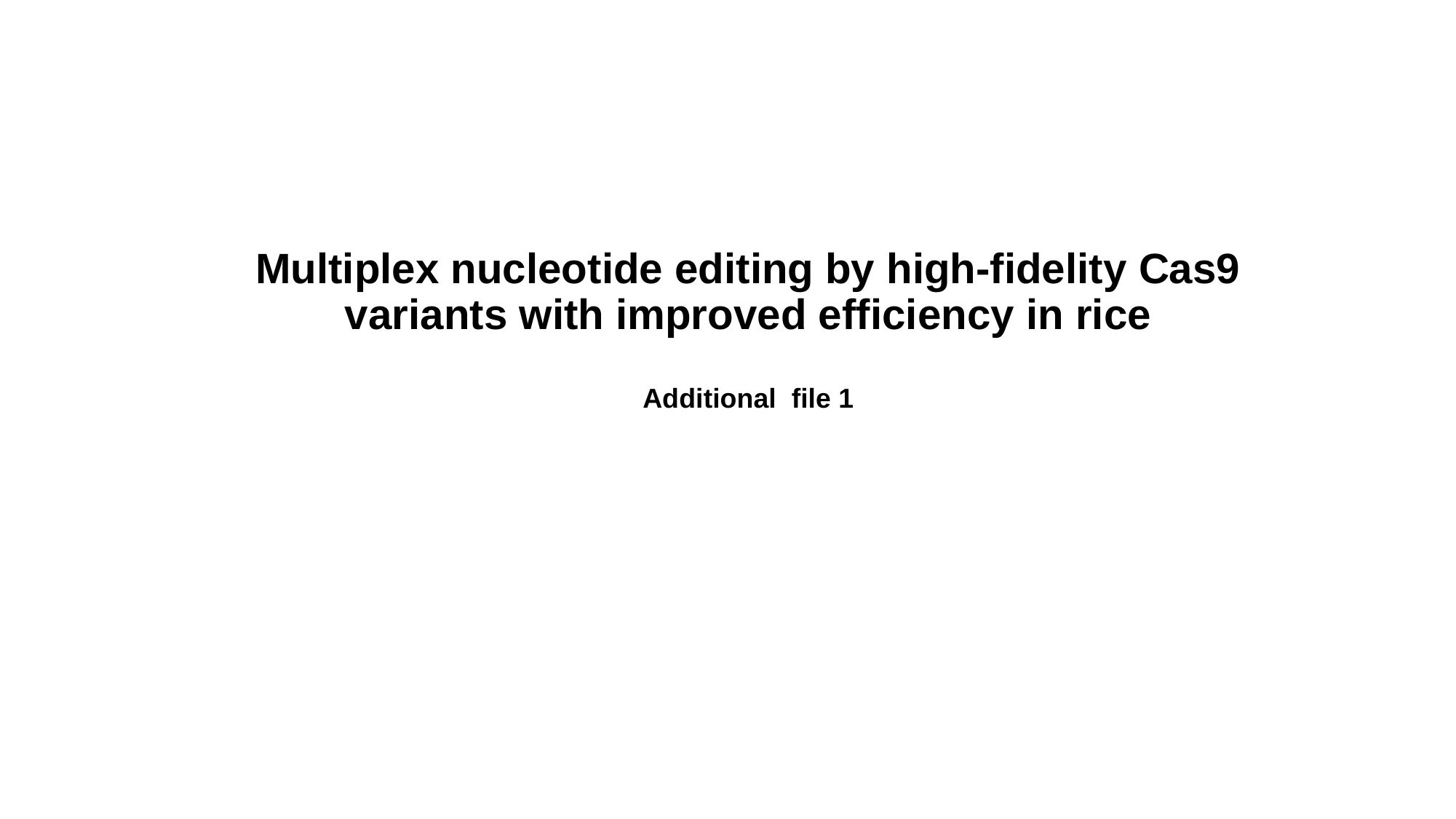

# Multiplex nucleotide editing by high-fidelity Cas9 variants with improved efficiency in rice Additional file 1

## Slide 2
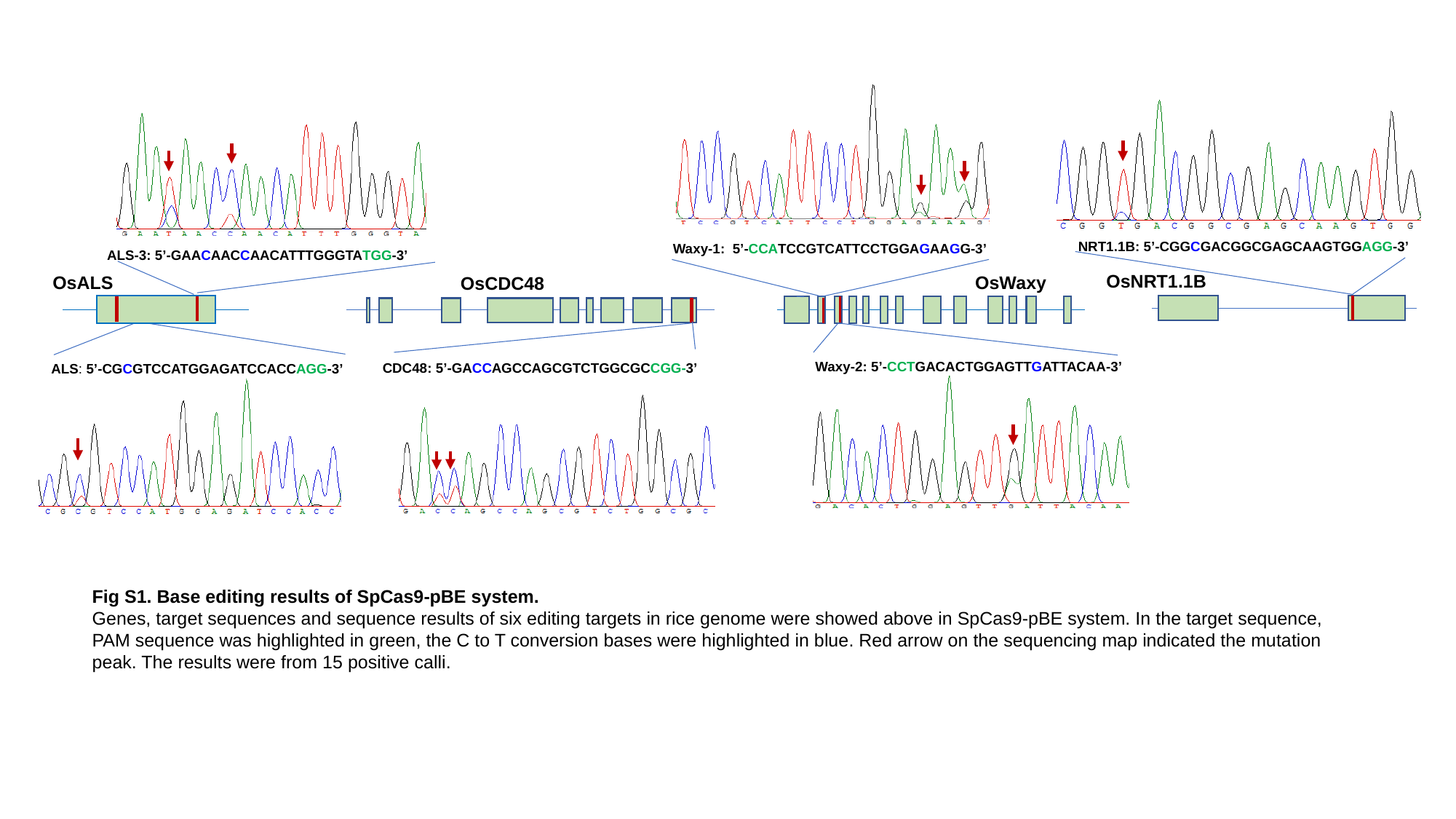

Waxy-1: 5’-CCATCCGTCATTCCTGGAGAAGG-3’
NRT1.1B: 5’-CGGCGACGGCGAGCAAGTGGAGG-3’
ALS-3: 5’-GAACAACCAACATTTGGGTATGG-3’
OsNRT1.1B
OsALS
OsWaxy
OsCDC48
CDC48: 5’-GACCAGCCAGCGTCTGGCGCCGG-3’
ALS: 5’-CGCGTCCATGGAGATCCACCAGG-3’
Waxy-2: 5’-CCTGACACTGGAGTTGATTACAA-3’
Fig S1. Base editing results of SpCas9-pBE system.
Genes, target sequences and sequence results of six editing targets in rice genome were showed above in SpCas9-pBE system. In the target sequence, PAM sequence was highlighted in green, the C to T conversion bases were highlighted in blue. Red arrow on the sequencing map indicated the mutation peak. The results were from 15 positive calli.

## Slide 3
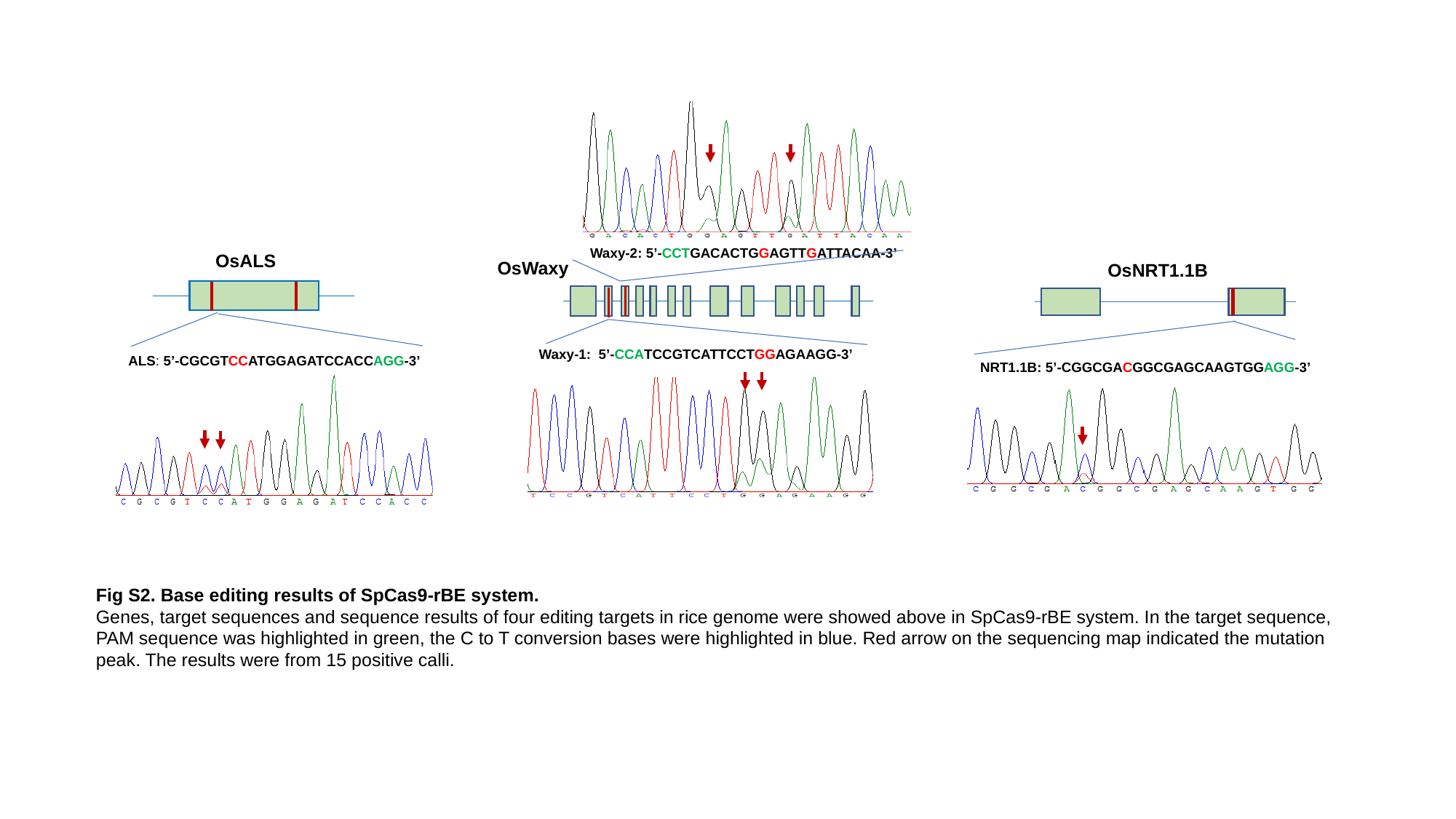

Waxy-2: 5’-CCTGACACTGGAGTTGATTACAA-3’
OsALS
ALS: 5’-CGCGTCCATGGAGATCCACCAGG-3’
OsWaxy
OsNRT1.1B
NRT1.1B: 5’-CGGCGACGGCGAGCAAGTGGAGG-3’
Waxy-1: 5’-CCATCCGTCATTCCTGGAGAAGG-3’
Fig S2. Base editing results of SpCas9-rBE system.
Genes, target sequences and sequence results of four editing targets in rice genome were showed above in SpCas9-rBE system. In the target sequence, PAM sequence was highlighted in green, the C to T conversion bases were highlighted in blue. Red arrow on the sequencing map indicated the mutation peak. The results were from 15 positive calli.

## Slide 4
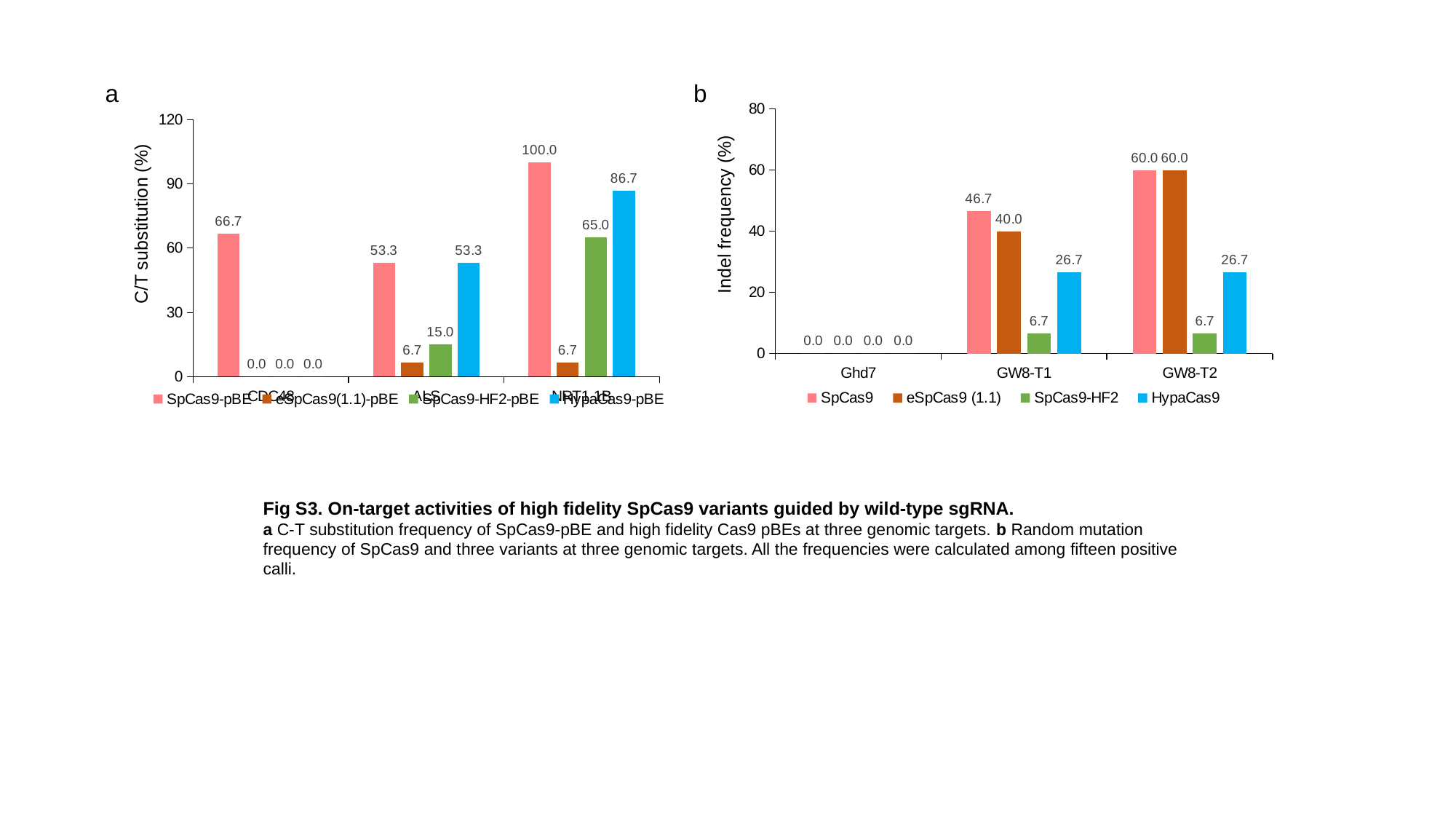

a
### Chart
| Category | SpCas9-pBE | eSpCas9(1.1)-pBE | SpCas9-HF2-pBE | HypaCas9-pBE |
|---|---|---|---|---|
| CDC48 | 66.7 | 0.0 | 0.0 | 0.0 |
| ALS | 53.300000000000004 | 6.7 | 15.0 | 53.300000000000004 |
| NRT1.1B | 100.0 | 6.7 | 65.0 | 86.7 |C/T substitution (%)
b
### Chart
| Category | SpCas9 | eSpCas9 (1.1) | SpCas9-HF2 | HypaCas9 |
|---|---|---|---|---|
| Ghd7 | 0.0 | 0.0 | 0.0 | 0.0 |
| GW8-T1 | 46.666666666666664 | 40.0 | 6.666666666666667 | 26.666666666666668 |
| GW8-T2 | 60.0 | 60.0 | 6.666666666666667 | 26.666666666666668 |Indel frequency (%)
Fig S3. On-target activities of high fidelity SpCas9 variants guided by wild-type sgRNA.
a C-T substitution frequency of SpCas9-pBE and high fidelity Cas9 pBEs at three genomic targets. b Random mutation frequency of SpCas9 and three variants at three genomic targets. All the frequencies were calculated among fifteen positive calli.

## Slide 5
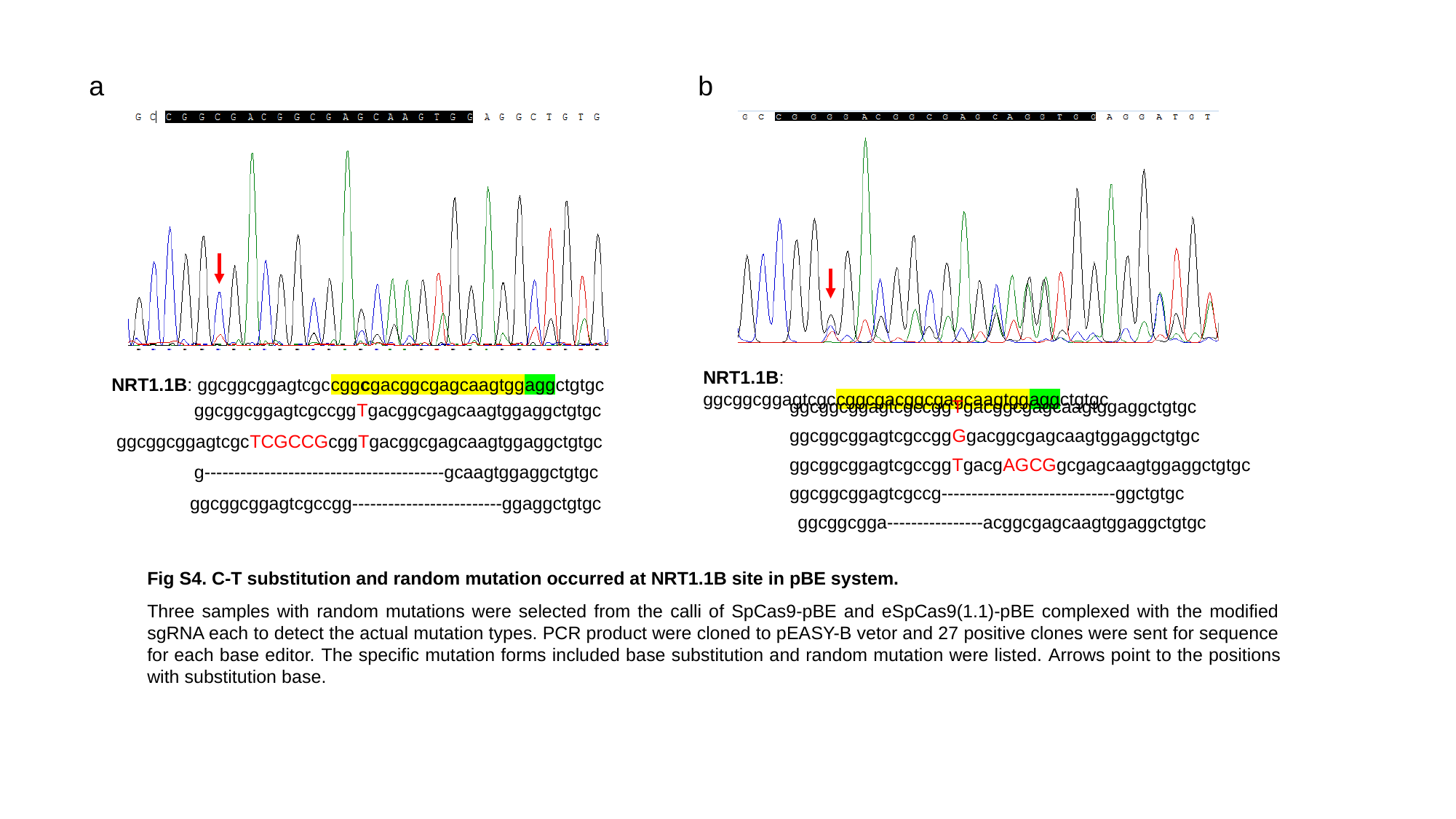

a
NRT1.1B: ggcggcggagtcgccggcgacggcgagcaagtggaggctgtgc
ggcggcggagtcgccggTgacggcgagcaagtggaggctgtgc
ggcggcggagtcgcTCGCCGcggTgacggcgagcaagtggaggctgtgc
g----------------------------------------gcaagtggaggctgtgc
ggcggcggagtcgccgg-------------------------ggaggctgtgc
b
NRT1.1B: ggcggcggagtcgccggcgacggcgagcaagtggaggctgtgc
ggcggcggagtcgccggTgacggcgagcaagtggaggctgtgc
ggcggcggagtcgccggGgacggcgagcaagtggaggctgtgc
ggcggcggagtcgccggTgacgAGCGgcgagcaagtggaggctgtgc
ggcggcggagtcgccg-----------------------------ggctgtgc
ggcggcgga----------------acggcgagcaagtggaggctgtgc
Fig S4. C-T substitution and random mutation occurred at NRT1.1B site in pBE system.
Three samples with random mutations were selected from the calli of SpCas9-pBE and eSpCas9(1.1)-pBE complexed with the modified sgRNA each to detect the actual mutation types. PCR product were cloned to pEASY-B vetor and 27 positive clones were sent for sequence for each base editor. The specific mutation forms included base substitution and random mutation were listed. Arrows point to the positions with substitution base.

## Slide 6
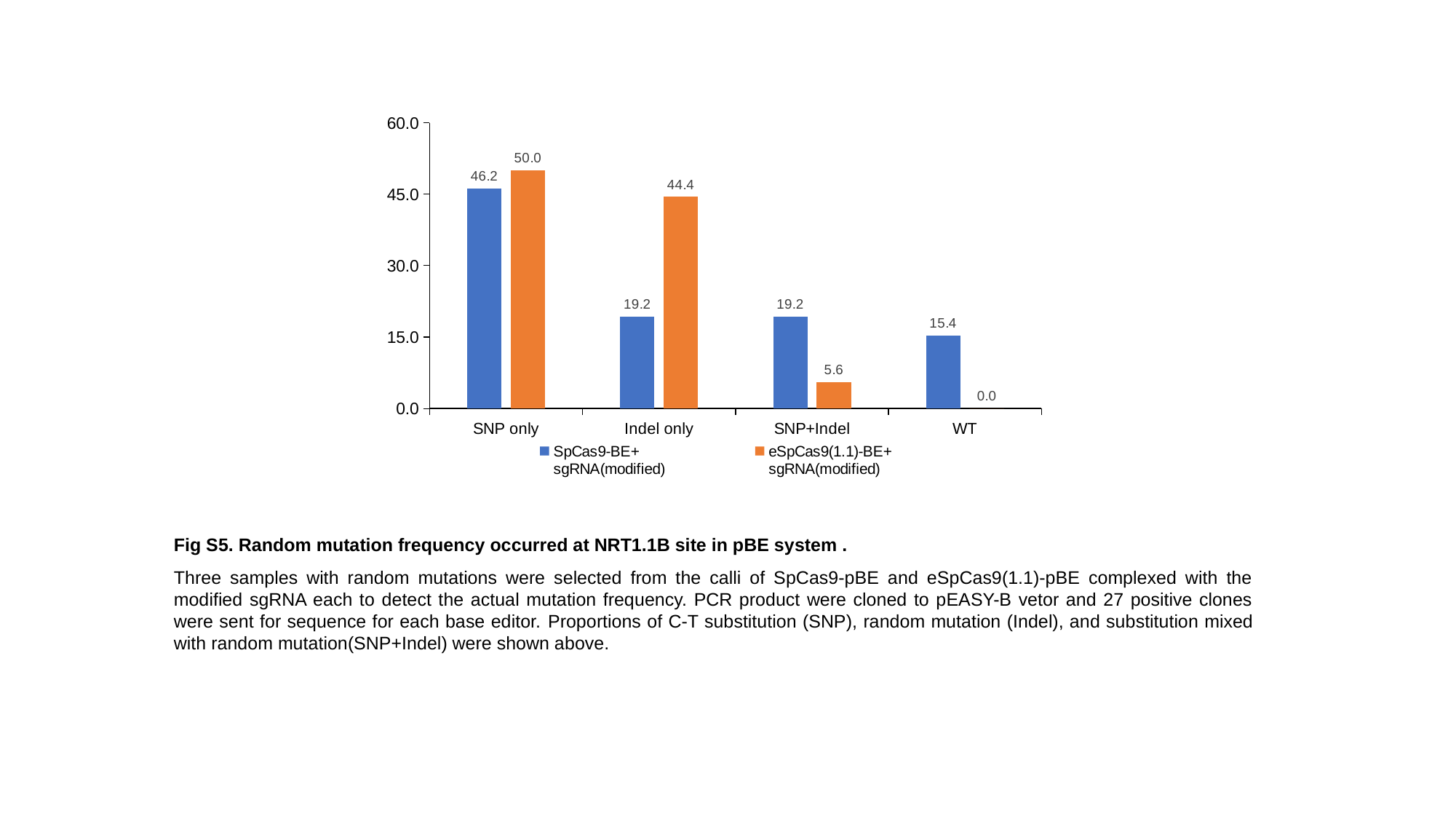

### Chart
| Category | SpCas9-BE+
sgRNA(modified) | eSpCas9(1.1)-BE+
sgRNA(modified) |
|---|---|---|
| SNP only | 46.15384615384615 | 50.0 |
| Indel only | 19.230769230769234 | 44.44444444444444 |
| SNP+Indel | 19.230769230769234 | 5.555555555555555 |
| WT | 15.384615384615385 | 0.0 |Fig S5. Random mutation frequency occurred at NRT1.1B site in pBE system .
Three samples with random mutations were selected from the calli of SpCas9-pBE and eSpCas9(1.1)-pBE complexed with the modified sgRNA each to detect the actual mutation frequency. PCR product were cloned to pEASY-B vetor and 27 positive clones were sent for sequence for each base editor. Proportions of C-T substitution (SNP), random mutation (Indel), and substitution mixed with random mutation(SNP+Indel) were shown above.

## Slide 7
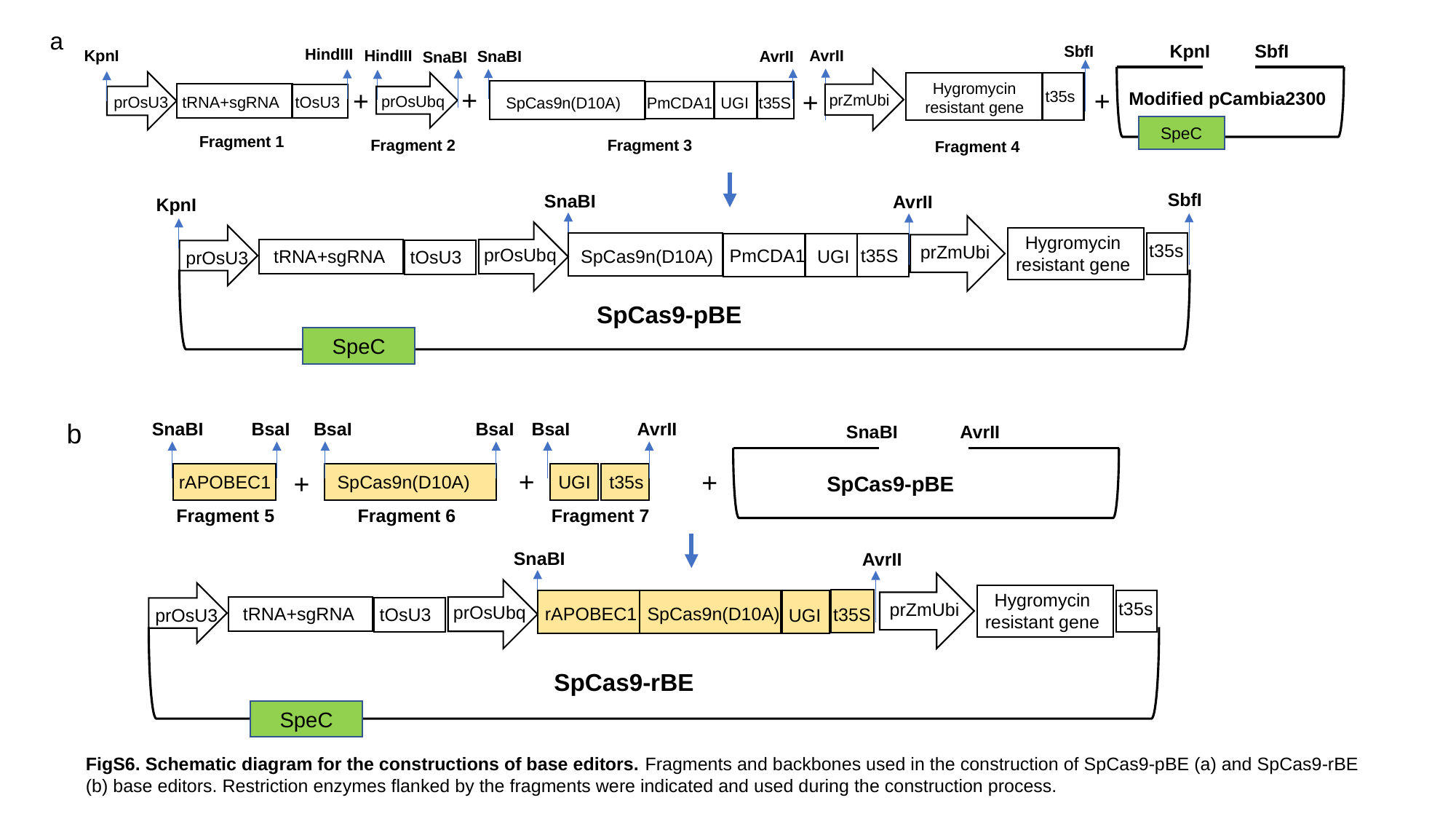

a
KpnI
SbfI
+
SbfI
AvrII
prZmUbi
Hygromycin resistant gene
t35s
Fragment 4
HindIII
KpnI
prOsU3
tRNA+sgRNA
tOsU3
Fragment 1
HindIII
SnaBI
prOsUbq
Fragment 2
SnaBI
AvrII
SpCas9n(D10A)
PmCDA1
UGI
t35S
Fragment 3
+
+
+
Modified pCambia2300
SpeC
SbfI
SnaBI
prOsUbq
AvrII
KpnI
prZmUbi
prOsU3
Hygromycin resistant gene
t35s
t35S
PmCDA1
UGI
SpCas9n(D10A)
tRNA+sgRNA
tOsU3
SpCas9-pBE
SpeC
b
SnaBI
BsaI
rAPOBEC1
BsaI
BsaI
SpCas9n(D10A)
BsaI
AvrII
UGI
t35s
+
+
SnaBI
AvrII
SpCas9-pBE
+
Fragment 5
Fragment 6
Fragment 7
SnaBI
prOsUbq
AvrII
prZmUbi
prOsU3
Hygromycin resistant gene
t35s
rAPOBEC1
tRNA+sgRNA
SpCas9n(D10A)
t35S
tOsU3
UGI
SpCas9-rBE
SpeC
FigS6. Schematic diagram for the constructions of base editors. Fragments and backbones used in the construction of SpCas9-pBE (a) and SpCas9-rBE (b) base editors. Restriction enzymes flanked by the fragments were indicated and used during the construction process.

## Slide 8
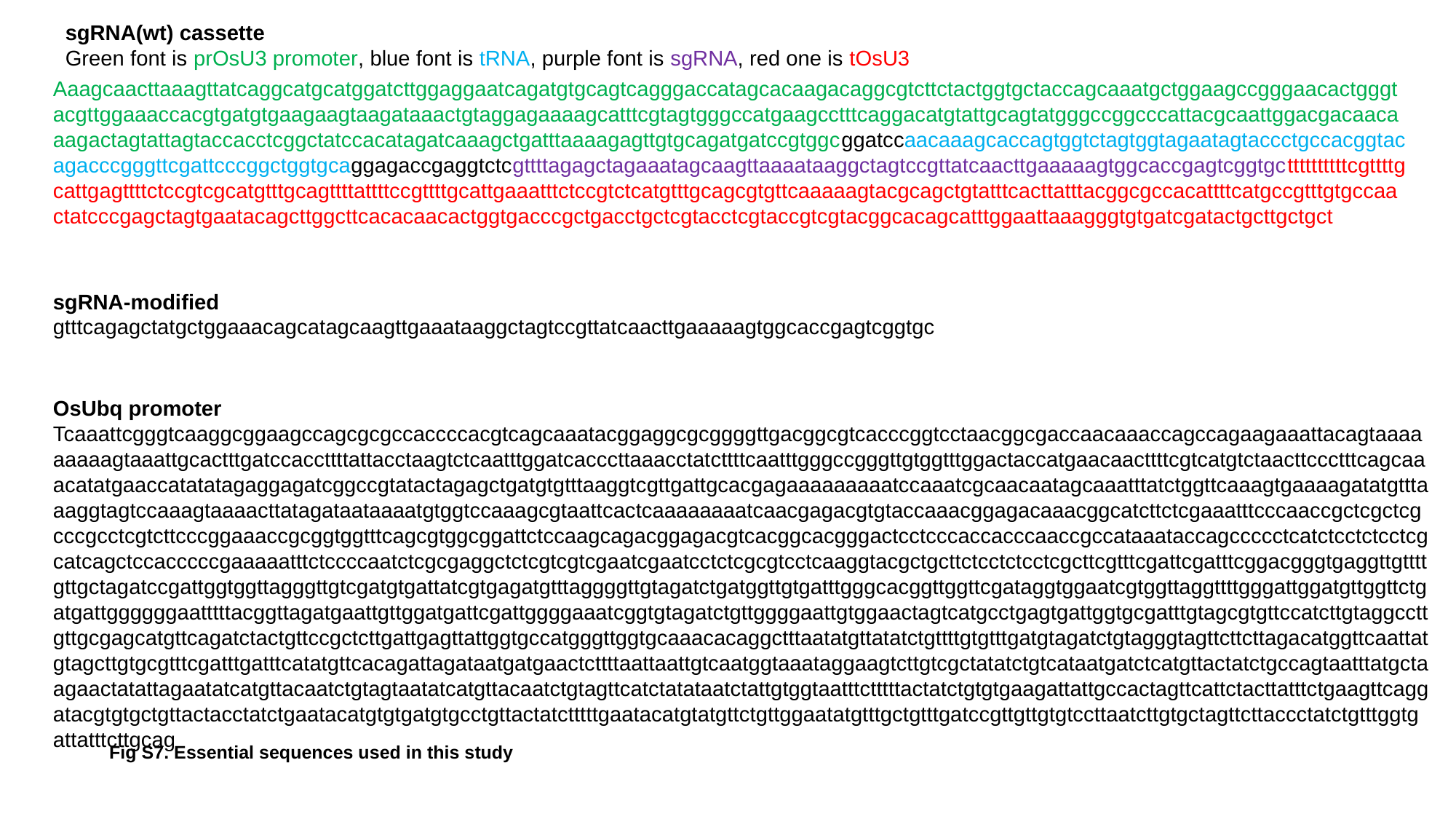

sgRNA(wt) cassette
Green font is prOsU3 promoter, blue font is tRNA, purple font is sgRNA, red one is tOsU3
Aaagcaacttaaagttatcaggcatgcatggatcttggaggaatcagatgtgcagtcagggaccatagcacaagacaggcgtcttctactggtgctaccagcaaatgctggaagccgggaacactgggtacgttggaaaccacgtgatgtgaagaagtaagataaactgtaggagaaaagcatttcgtagtgggccatgaagcctttcaggacatgtattgcagtatgggccggcccattacgcaattggacgacaacaaagactagtattagtaccacctcggctatccacatagatcaaagctgatttaaaagagttgtgcagatgatccgtggcggatccaacaaagcaccagtggtctagtggtagaatagtaccctgccacggtacagacccgggttcgattcccggctggtgcaggagaccgaggtctcgttttagagctagaaatagcaagttaaaataaggctagtccgttatcaacttgaaaaagtggcaccgagtcggtgcttttttttttcgttttgcattgagttttctccgtcgcatgtttgcagttttattttccgttttgcattgaaatttctccgtctcatgtttgcagcgtgttcaaaaagtacgcagctgtatttcacttatttacggcgccacattttcatgccgtttgtgccaactatcccgagctagtgaatacagcttggcttcacacaacactggtgacccgctgacctgctcgtacctcgtaccgtcgtacggcacagcatttggaattaaagggtgtgatcgatactgcttgctgct
sgRNA-modified
gtttcagagctatgctggaaacagcatagcaagttgaaataaggctagtccgttatcaacttgaaaaagtggcaccgagtcggtgc
OsUbq promoter
Tcaaattcgggtcaaggcggaagccagcgcgccaccccacgtcagcaaatacggaggcgcggggttgacggcgtcacccggtcctaacggcgaccaacaaaccagccagaagaaattacagtaaaaaaaaagtaaattgcactttgatccaccttttattacctaagtctcaatttggatcacccttaaacctatcttttcaatttgggccgggttgtggtttggactaccatgaacaacttttcgtcatgtctaacttccctttcagcaaacatatgaaccatatatagaggagatcggccgtatactagagctgatgtgtttaaggtcgttgattgcacgagaaaaaaaaatccaaatcgcaacaatagcaaatttatctggttcaaagtgaaaagatatgtttaaaggtagtccaaagtaaaacttatagataataaaatgtggtccaaagcgtaattcactcaaaaaaaatcaacgagacgtgtaccaaacggagacaaacggcatcttctcgaaatttcccaaccgctcgctcgcccgcctcgtcttcccggaaaccgcggtggtttcagcgtggcggattctccaagcagacggagacgtcacggcacgggactcctcccaccacccaaccgccataaataccagccccctcatctcctctcctcgcatcagctccacccccgaaaaatttctccccaatctcgcgaggctctcgtcgtcgaatcgaatcctctcgcgtcctcaaggtacgctgcttctcctctcctcgcttcgtttcgattcgatttcggacgggtgaggttgttttgttgctagatccgattggtggttagggttgtcgatgtgattatcgtgagatgtttaggggttgtagatctgatggttgtgatttgggcacggttggttcgataggtggaatcgtggttaggttttgggattggatgttggttctgatgattggggggaatttttacggttagatgaattgttggatgattcgattggggaaatcggtgtagatctgttggggaattgtggaactagtcatgcctgagtgattggtgcgatttgtagcgtgttccatcttgtaggccttgttgcgagcatgttcagatctactgttccgctcttgattgagttattggtgccatgggttggtgcaaacacaggctttaatatgttatatctgttttgtgtttgatgtagatctgtagggtagttcttcttagacatggttcaattatgtagcttgtgcgtttcgatttgatttcatatgttcacagattagataatgatgaactcttttaattaattgtcaatggtaaataggaagtcttgtcgctatatctgtcataatgatctcatgttactatctgccagtaatttatgctaagaactatattagaatatcatgttacaatctgtagtaatatcatgttacaatctgtagttcatctatataatctattgtggtaatttctttttactatctgtgtgaagattattgccactagttcattctacttatttctgaagttcaggatacgtgtgctgttactacctatctgaatacatgtgtgatgtgcctgttactatctttttgaatacatgtatgttctgttggaatatgtttgctgtttgatccgttgttgtgtccttaatcttgtgctagttcttaccctatctgtttggtgattatttcttgcag
Fig S7. Essential sequences used in this study

## Slide 9
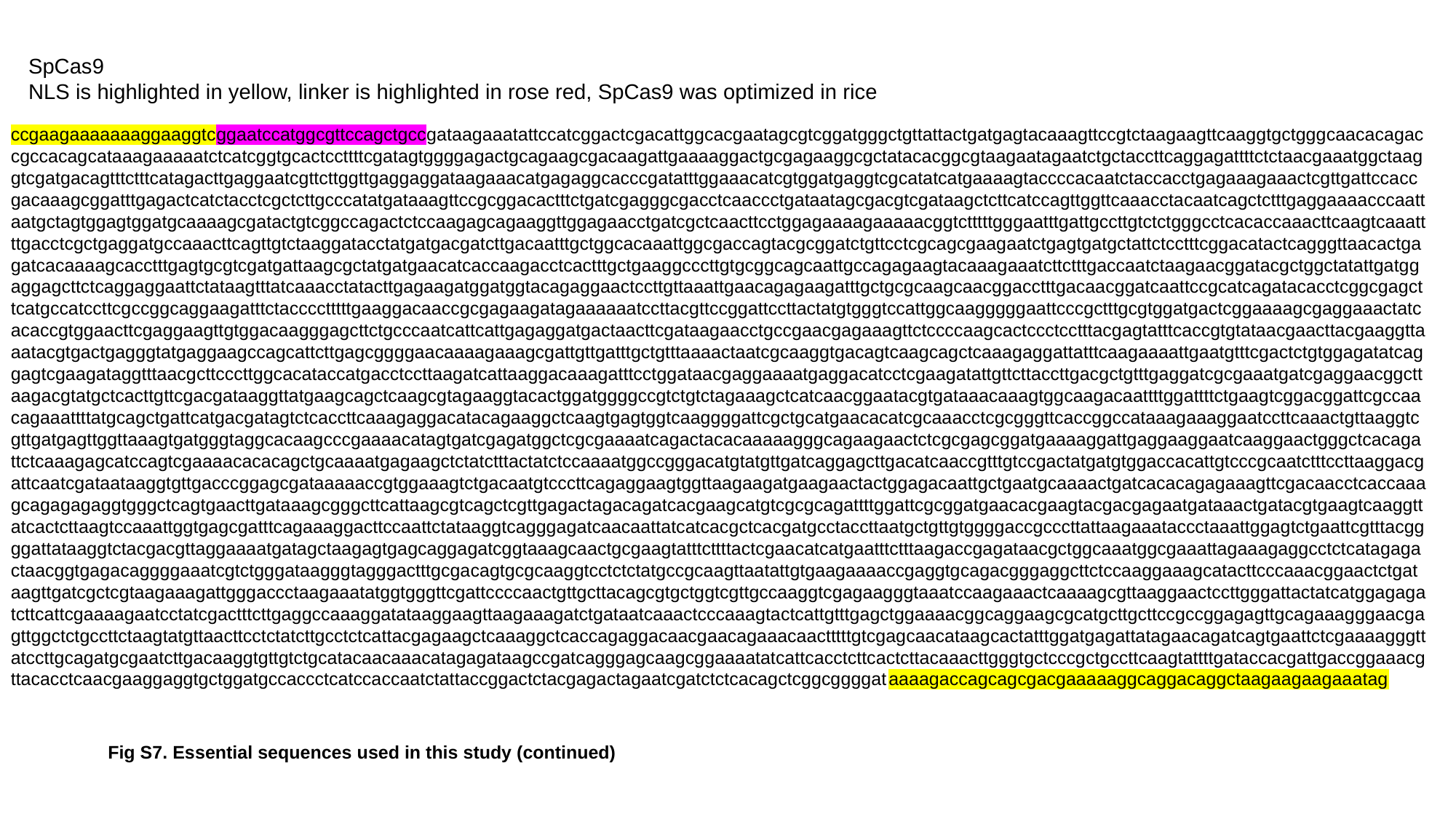

SpCas9
NLS is highlighted in yellow, linker is highlighted in rose red, SpCas9 was optimized in rice
ccgaagaaaaaaaggaaggtcggaatccatggcgttccagctgccgataagaaatattccatcggactcgacattggcacgaatagcgtcggatgggctgttattactgatgagtacaaagttccgtctaagaagttcaaggtgctgggcaacacagaccgccacagcataaagaaaaatctcatcggtgcactccttttcgatagtggggagactgcagaagcgacaagattgaaaaggactgcgagaaggcgctatacacggcgtaagaatagaatctgctaccttcaggagattttctctaacgaaatggctaaggtcgatgacagtttctttcatagacttgaggaatcgttcttggttgaggaggataagaaacatgagaggcacccgatatttggaaacatcgtggatgaggtcgcatatcatgaaaagtaccccacaatctaccacctgagaaagaaactcgttgattccaccgacaaagcggatttgagactcatctacctcgctcttgcccatatgataaagttccgcggacactttctgatcgagggcgacctcaaccctgataatagcgacgtcgataagctcttcatccagttggttcaaacctacaatcagctctttgaggaaaacccaattaatgctagtggagtggatgcaaaagcgatactgtcggccagactctccaagagcagaaggttggagaacctgatcgctcaacttcctggagaaaagaaaaacggtctttttgggaatttgattgccttgtctctgggcctcacaccaaacttcaagtcaaattttgacctcgctgaggatgccaaacttcagttgtctaaggatacctatgatgacgatcttgacaatttgctggcacaaattggcgaccagtacgcggatctgttcctcgcagcgaagaatctgagtgatgctattctcctttcggacatactcagggttaacactgagatcacaaaagcacctttgagtgcgtcgatgattaagcgctatgatgaacatcaccaagacctcactttgctgaaggcccttgtgcggcagcaattgccagagaagtacaaagaaatcttctttgaccaatctaagaacggatacgctggctatattgatggaggagcttctcaggaggaattctataagtttatcaaacctatacttgagaagatggatggtacagaggaactccttgttaaattgaacagagaagatttgctgcgcaagcaacggacctttgacaacggatcaattccgcatcagatacacctcggcgagcttcatgccatccttcgccggcaggaagatttctacccctttttgaaggacaaccgcgagaagatagaaaaaatccttacgttccggattccttactatgtgggtccattggcaagggggaattcccgctttgcgtggatgactcggaaaagcgaggaaactatcacaccgtggaacttcgaggaagttgtggacaagggagcttctgcccaatcattcattgagaggatgactaacttcgataagaacctgccgaacgagaaagttctccccaagcactccctcctttacgagtatttcaccgtgtataacgaacttacgaaggttaaatacgtgactgagggtatgaggaagccagcattcttgagcggggaacaaaagaaagcgattgttgatttgctgtttaaaactaatcgcaaggtgacagtcaagcagctcaaagaggattatttcaagaaaattgaatgtttcgactctgtggagatatcaggagtcgaagataggtttaacgcttcccttggcacataccatgacctccttaagatcattaaggacaaagatttcctggataacgaggaaaatgaggacatcctcgaagatattgttcttaccttgacgctgtttgaggatcgcgaaatgatcgaggaacggcttaagacgtatgctcacttgttcgacgataaggttatgaagcagctcaagcgtagaaggtacactggatggggccgtctgtctagaaagctcatcaacggaatacgtgataaacaaagtggcaagacaattttggattttctgaagtcggacggattcgccaacagaaattttatgcagctgattcatgacgatagtctcaccttcaaagaggacatacagaaggctcaagtgagtggtcaaggggattcgctgcatgaacacatcgcaaacctcgcgggttcaccggccataaagaaaggaatccttcaaactgttaaggtcgttgatgagttggttaaagtgatgggtaggcacaagcccgaaaacatagtgatcgagatggctcgcgaaaatcagactacacaaaaagggcagaagaactctcgcgagcggatgaaaaggattgaggaaggaatcaaggaactgggctcacagattctcaaagagcatccagtcgaaaacacacagctgcaaaatgagaagctctatctttactatctccaaaatggccgggacatgtatgttgatcaggagcttgacatcaaccgtttgtccgactatgatgtggaccacattgtcccgcaatctttccttaaggacgattcaatcgataataaggtgttgacccggagcgataaaaaccgtggaaagtctgacaatgtcccttcagaggaagtggttaagaagatgaagaactactggagacaattgctgaatgcaaaactgatcacacagagaaagttcgacaacctcaccaaagcagagagaggtgggctcagtgaacttgataaagcgggcttcattaagcgtcagctcgttgagactagacagatcacgaagcatgtcgcgcagattttggattcgcggatgaacacgaagtacgacgagaatgataaactgatacgtgaagtcaaggttatcactcttaagtccaaattggtgagcgatttcagaaaggacttccaattctataaggtcagggagatcaacaattatcatcacgctcacgatgcctaccttaatgctgttgtggggaccgcccttattaagaaataccctaaattggagtctgaattcgtttacggggattataaggtctacgacgttaggaaaatgatagctaagagtgagcaggagatcggtaaagcaactgcgaagtatttcttttactcgaacatcatgaatttctttaagaccgagataacgctggcaaatggcgaaattagaaagaggcctctcatagagactaacggtgagacaggggaaatcgtctgggataagggtagggactttgcgacagtgcgcaaggtcctctctatgccgcaagttaatattgtgaagaaaaccgaggtgcagacgggaggcttctccaaggaaagcatacttcccaaacggaactctgataagttgatcgctcgtaagaaagattgggaccctaagaaatatggtgggttcgattccccaactgttgcttacagcgtgctggtcgttgccaaggtcgagaagggtaaatccaagaaactcaaaagcgttaaggaactccttgggattactatcatggagagatcttcattcgaaaagaatcctatcgactttcttgaggccaaaggatataaggaagttaagaaagatctgataatcaaactcccaaagtactcattgtttgagctggaaaacggcaggaagcgcatgcttgcttccgccggagagttgcagaaagggaacgagttggctctgccttctaagtatgttaacttcctctatcttgcctctcattacgagaagctcaaaggctcaccagaggacaacgaacagaaacaactttttgtcgagcaacataagcactatttggatgagattatagaacagatcagtgaattctcgaaaagggttatccttgcagatgcgaatcttgacaaggtgttgtctgcatacaacaaacatagagataagccgatcagggagcaagcggaaaatatcattcacctcttcactcttacaaacttgggtgctcccgctgccttcaagtattttgataccacgattgaccggaaacgttacacctcaacgaaggaggtgctggatgccaccctcatccaccaatctattaccggactctacgagactagaatcgatctctcacagctcggcggggataaaagaccagcagcgacgaaaaaggcaggacaggctaagaagaagaaatag
Fig S7. Essential sequences used in this study (continued)

## Slide 10
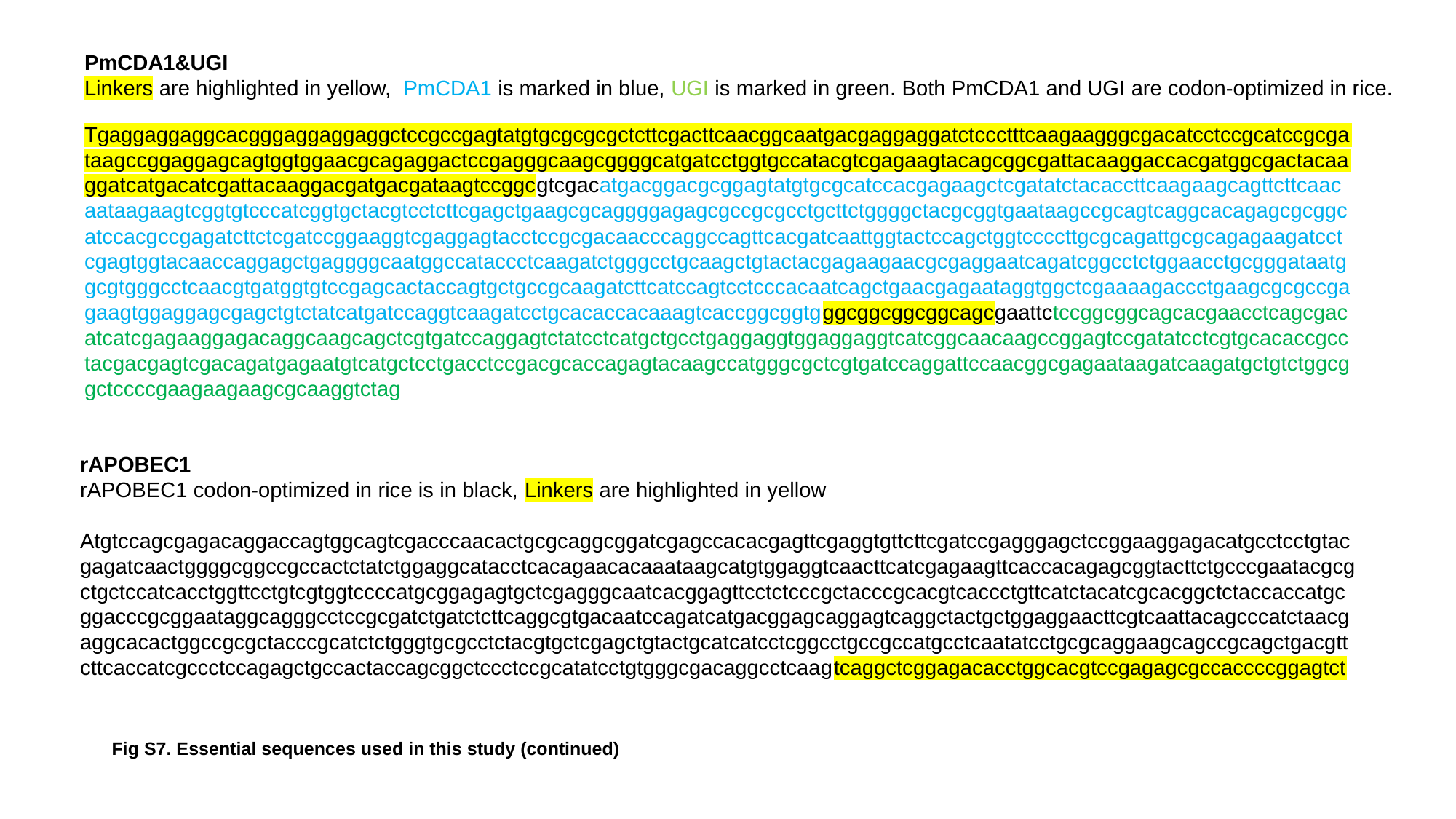

PmCDA1&UGI
Linkers are highlighted in yellow, PmCDA1 is marked in blue, UGI is marked in green. Both PmCDA1 and UGI are codon-optimized in rice.
Tgaggaggaggcacgggaggaggaggctccgccgagtatgtgcgcgcgctcttcgacttcaacggcaatgacgaggaggatctccctttcaagaagggcgacatcctccgcatccgcgataagccggaggagcagtggtggaacgcagaggactccgagggcaagcggggcatgatcctggtgccatacgtcgagaagtacagcggcgattacaaggaccacgatggcgactacaaggatcatgacatcgattacaaggacgatgacgataagtccggcgtcgacatgacggacgcggagtatgtgcgcatccacgagaagctcgatatctacaccttcaagaagcagttcttcaacaataagaagtcggtgtcccatcggtgctacgtcctcttcgagctgaagcgcaggggagagcgccgcgcctgcttctggggctacgcggtgaataagccgcagtcaggcacagagcgcggcatccacgccgagatcttctcgatccggaaggtcgaggagtacctccgcgacaacccaggccagttcacgatcaattggtactccagctggtccccttgcgcagattgcgcagagaagatcctcgagtggtacaaccaggagctgaggggcaatggccataccctcaagatctgggcctgcaagctgtactacgagaagaacgcgaggaatcagatcggcctctggaacctgcgggataatggcgtgggcctcaacgtgatggtgtccgagcactaccagtgctgccgcaagatcttcatccagtcctcccacaatcagctgaacgagaataggtggctcgaaaagaccctgaagcgcgccgagaagtggaggagcgagctgtctatcatgatccaggtcaagatcctgcacaccacaaagtcaccggcggtgggcggcggcggcagcgaattctccggcggcagcacgaacctcagcgacatcatcgagaaggagacaggcaagcagctcgtgatccaggagtctatcctcatgctgcctgaggaggtggaggaggtcatcggcaacaagccggagtccgatatcctcgtgcacaccgcctacgacgagtcgacagatgagaatgtcatgctcctgacctccgacgcaccagagtacaagccatgggcgctcgtgatccaggattccaacggcgagaataagatcaagatgctgtctggcggctccccgaagaagaagcgcaaggtctag
rAPOBEC1
rAPOBEC1 codon-optimized in rice is in black, Linkers are highlighted in yellow
Atgtccagcgagacaggaccagtggcagtcgacccaacactgcgcaggcggatcgagccacacgagttcgaggtgttcttcgatccgagggagctccggaaggagacatgcctcctgtacgagatcaactggggcggccgccactctatctggaggcatacctcacagaacacaaataagcatgtggaggtcaacttcatcgagaagttcaccacagagcggtacttctgcccgaatacgcgctgctccatcacctggttcctgtcgtggtccccatgcggagagtgctcgagggcaatcacggagttcctctcccgctacccgcacgtcaccctgttcatctacatcgcacggctctaccaccatgcggacccgcggaataggcagggcctccgcgatctgatctcttcaggcgtgacaatccagatcatgacggagcaggagtcaggctactgctggaggaacttcgtcaattacagcccatctaacgaggcacactggccgcgctacccgcatctctgggtgcgcctctacgtgctcgagctgtactgcatcatcctcggcctgccgccatgcctcaatatcctgcgcaggaagcagccgcagctgacgttcttcaccatcgccctccagagctgccactaccagcggctccctccgcatatcctgtgggcgacaggcctcaagtcaggctcggagacacctggcacgtccgagagcgccaccccggagtct
Fig S7. Essential sequences used in this study (continued)

## Slide 11
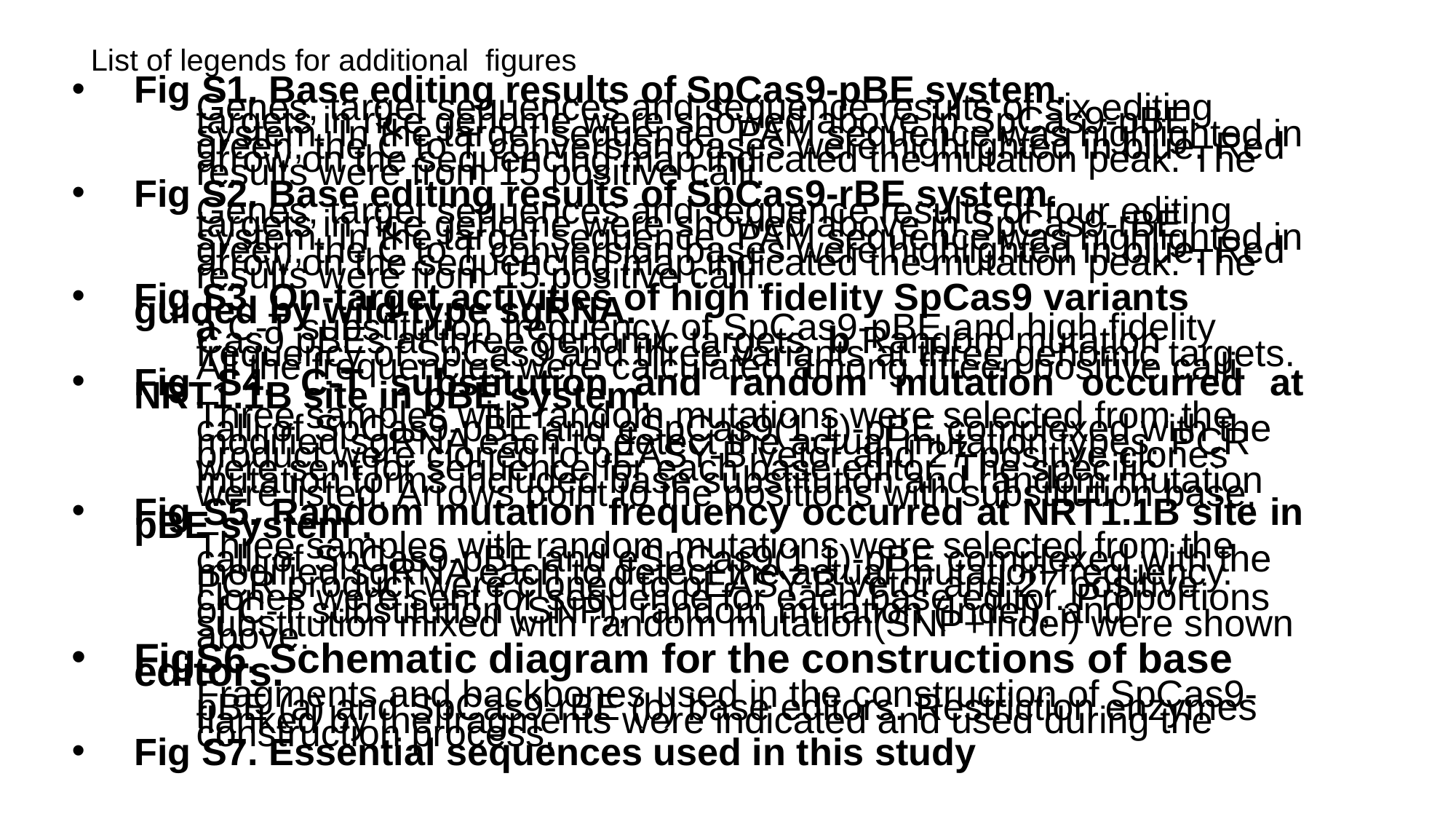

# List of legends for additional figures
Fig S1. Base editing results of SpCas9-pBE system.
Genes, target sequences and sequence results of six editing targets in rice genome were showed above in SpCas9-pBE system. In the target sequence, PAM sequence was highlighted in green, the C to T conversion bases were highlighted in blue. Red arrow on the sequencing map indicated the mutation peak. The results were from 15 positive calli.
Fig S2. Base editing results of SpCas9-rBE system.
Genes, target sequences and sequence results of four editing targets in rice genome were showed above in SpCas9-rBE system. In the target sequence, PAM sequence was highlighted in green, the C to T conversion bases were highlighted in blue. Red arrow on the sequencing map indicated the mutation peak. The results were from 15 positive calli.
Fig S3. On-target activities of high fidelity SpCas9 variants guided by wild-type sgRNA.
a C-T substitution frequency of SpCas9-pBE and high fidelity Cas9 pBEs at three genomic targets. b Random mutation frequency of SpCas9 and three variants at three genomic targets. All the frequencies were calculated among fifteen positive calli.
Fig S4. C-T substitution and random mutation occurred at NRT1.1B site in pBE system.
Three samples with random mutations were selected from the calli of SpCas9-pBE and eSpCas9(1.1)-pBE complexed with the modified sgRNA each to detect the actual mutation types. PCR product were cloned to pEASY-B vetor and 27 positive clones were sent for sequence for each base editor. The specific mutation forms included base substitution and random mutation were listed. Arrows point to the positions with substitution base.
Fig S5. Random mutation frequency occurred at NRT1.1B site in pBE system .
Three samples with random mutations were selected from the calli of SpCas9-pBE and eSpCas9(1.1)-pBE complexed with the modified sgRNA each to detect the actual mutation frequency. PCR product were cloned to pEASY-B vetor and 27 positive clones were sent for sequence for each base editor. Proportions of C-T substitution (SNP), random mutation (Indel), and substitution mixed with random mutation(SNP+Indel) were shown above.
FigS6. Schematic diagram for the constructions of base editors.
Fragments and backbones used in the construction of SpCas9-pBE (a) and SpCas9-rBE (b) base editors. Restriction enzymes flanked by the fragments were indicated and used during the construction process.
Fig S7. Essential sequences used in this study
