# Supplementary material for: Multiplex nucleotide editing by high-fidelity Cas9 variants with improved efficiency in rice
Source: BMC Plant Biol. 2019 Nov 21;19:511. doi: 10.1186/s12870-019-2131-1 (PMC6873407; doi:10.1186/s12870-019-2131-1)
Supplement: Supplementary file 2 — Additional file 2 : Table S1. Comparation of base editing efficiency in our study with reported results. Table S2. Base editing preference of SpCas9-rBE and SpCas9-pBE in rice. Table S3. Precise base editing preferences and frequencies in all SpCas9 base editors on OsCDC48, OsALS and OsNRT1.1B sites. Table S4. The frequency of random mutation occurred during precise base editing on OsCDC48, OsALS and OsNRT1.1B sites in all SpCas9 base editors. Table S5. Potential off-target sites in rice genomics for base editing targets. Table S6. Potential off-target sites in rice genomics for random mutation targets. Table S7. Primers used in this study. [file 12870_2019_2131_MOESM2_ESM.docx]

**Additional file 2, supplementary tables**

**Table S1.** Comparation of base editing efficiency in our study with reported results.

| **Target site** | **Target sequence** | **rBE efficiency in our study** | **pBE efficiency in our study** | **Reported efficiency** | **Citation** |
| --- | --- | --- | --- | --- | --- |
| CDC48 | **g**accagccagcgtctggcgc | 0 | 66.7% | 4-6% in protoplast, 43.48% in T0 plants (rAPOBEC1) | Zong Y et al., 2017 |
| ALS | **c**gcgtccatggagatccacc | 5% | 53.3% | 3.41% in callus ( PmCDA1) | Shimatani Z et al., 2017 |
| NRT1.1B | **c**ggcgacggcgagcaagtgg | 10% | 100% | 1.4-11.5% in callus, No mutation in T0 plants (rAPOBEC1) | Lu Y, Zhu JK., 2017 |
| Waxy-1 | **c**cttctccaggaatgacgga | 20% | 60% | None | None |
| Waxy-2 | **t**tgtaatcaactccagtgtc | 10% | 5% | None | None |
| ALS-2 | **c**ctcatgaacattcaggagc | 0 | 5% | None | None |
| ALS-3 | **g**aacaaccaacatttgggta | 0 | 25% | None | None |

**Table S2.** Base editing preference of SpCas9-rBE and SpCas9-pBE in rice.

| **CDC48** | 1 | 2 | 3 | 4 | 5 | 6 | 7 | 8 | 9 | 10 | 11 | 12 | 13 | 14 | 15 | 16 | 17 | 18 | 19 | 20 |
| --- | --- | --- | --- | --- | --- | --- | --- | --- | --- | --- | --- | --- | --- | --- | --- | --- | --- | --- | --- | --- |
| **sgRNA** | G | A | C | C | A | G | C | C | A | G | C | G | T | C | T | G | G | C | G | C |
| **SpCas9-rBE** |  |  |  |  |  |  |  |  |  |  |  |  |  |  |  |  |  |  |  |  |
| **SpCas9-pBE** |  |  | 66.7 | 66.7 |  |  |  |  |  |  |  |  |  |  |  |  |  |  |  |  |

| **ALS** | 1 | 2 | 3 | 4 | 5 | 6 | 7 | 8 | 9 | 1000 | 11 | 12 | 13 | 14 | 15 | 16 | 17 | 18 | 19 | 20 |
| --- | --- | --- | --- | --- | --- | --- | --- | --- | --- | --- | --- | --- | --- | --- | --- | --- | --- | --- | --- | --- |
| **sgRNA** | C | G | C | G | T | C | C | A | T | G | G | A | G | A | T | C | C | A | C | C |
| **SpCas9-rBE** |  |  |  |  |  | 5 | 5 |  |  |  |  |  |  |  |  |  |  |  |  |  |
| **SpCas9-pBE** |  |  | 533 |  |  |  |  |  |  |  |  |  |  |  |  |  |  |  |  |  |

| **NRT1.1B** | 1 | 2 | 3 | 4 | 5 | 6 | 7 | 8 | 9 | 10 | 11 | 12 | 13 | 14 | 15 | 16 | 17 | 18 | 19 | 20 |
| --- | --- | --- | --- | --- | --- | --- | --- | --- | --- | --- | --- | --- | --- | --- | --- | --- | --- | --- | --- | --- |
| **sgRNA** | C | G | G | C | G | A | C | G | G | C | G | A | G | C | A | A | G | T | G | G |
| **SpCas9-rBE** |  |  |  |  |  |  | 10 |  |  |  |  |  |  |  |  |  |  |  |  |  |
| **SpCas9-pBE** | 25 |  |  | 100 |  |  |  |  |  |  |  |  |  |  |  |  |  |  |  |  |

| **Waxy-1** | 1 | 2 | 3 | 4 | 5 | 6 | 7 | 8 | 9 | 10 | 11 | 12 | 13 | 14 | 15 | 16 | 17 | 18 | 19 | 20 |
| --- | --- | --- | --- | --- | --- | --- | --- | --- | --- | --- | --- | --- | --- | --- | --- | --- | --- | --- | --- | --- |
| **sgRNA** | C | C | T | T | C | T | C | C | A | G | G | A | A | T | G | A | C | G | G | A |
| **SpCas9-rBE** |  |  |  |  |  |  | 20 | 15 |  |  |  |  |  |  |  |  |  |  |  |  |
| **SpCas9-pBE** | 15 | 60 |  |  | 45 |  | 5 |  |  |  |  |  |  |  |  |  |  |  |  |  |

| **Waxy-2** | 1 | 2 | 3 | 4 | 5 | 6 | 7 | 8 | 9 | 10 | 11 | 12 | 13 | 14 | 15 | 16 | 17 | 18 | 19 | 20 |
| --- | --- | --- | --- | --- | --- | --- | --- | --- | --- | --- | --- | --- | --- | --- | --- | --- | --- | --- | --- | --- |
| **sgRNA** | T | T | G | T | A | A | T | C | A | A | C | T | C | C | A | G | T | G | T | C |
| **SpCas9-rBE** |  |  |  |  |  |  |  | 10 |  |  |  |  | 10 |  |  |  |  |  |  |  |
| **SpCas9-pBE** |  |  |  |  |  |  |  | 5 |  |  |  |  |  |  |  |  |  |  |  |  |

**Table S3.** Precise base editing preferences and frequencies in all SpCas9 base editors on OsCDC48, OsALS and OsNRT1.1B sites.

|  | **CDC48** | 1 | 2 | 3 | 4 | 5 | 6 | 7 | 8 | 9 | 10 | 11 | 12 | 13 | 14 | 15 | 16 | 17 | 18 | 19 | 20 |
| --- | --- | --- | --- | --- | --- | --- | --- | --- | --- | --- | --- | --- | --- | --- | --- | --- | --- | --- | --- | --- | --- |
| **Base Editor** | **sgRNA** | G | A | C | C | A | G | C | C | A | G | C | G | T | C | T | G | G | C | G | C |
| SpCas9-pBE | WT |  |  | 60 | 66.7 |  |  |  |  |  |  |  |  |  |  |  |  |  |  |  |  |
|  | modified |  |  | 66.7 | 73.3 |  |  |  |  |  |  |  |  |  |  |  |  |  |  |  |  |
| eSpCas9(1.1)-pBE | WT |  |  | 0 |  |  |  |  |  |  |  |  |  |  |  |  |  |  |  |  |  |
|  | modified |  |  | 40 | 73.3 |  |  |  |  |  |  |  |  |  |  |  |  |  |  |  |  |
| SpCas9-HF2-pBE | WT |  |  | 0 | 0 |  |  |  |  |  |  |  |  |  |  |  |  |  |  |  |  |
|  | modified |  |  | 0 | 0 |  |  |  |  |  |  |  |  |  |  |  |  |  |  |  |  |
| HypaCas9-pBE | WT |  |  | 0 | 0 |  |  |  |  |  |  |  |  |  |  |  |  |  |  |  |  |
|  | modified |  |  | 40 | 40 |  |  |  |  |  |  |  |  |  |  |  |  |  |  |  |  |

|  | **ALS** | 1 | 2 | 3 | 4 | 5 | 6 | 7 | 8 | 9 | 10 | 11 | 12 | 13 | 14 | 15 | 16 | 17 | 18 | 19 | 20 |
| --- | --- | --- | --- | --- | --- | --- | --- | --- | --- | --- | --- | --- | --- | --- | --- | --- | --- | --- | --- | --- | --- |
| **Base Editor** | **sgRNA** | C | G | C | G | T | C | C | A | T | G | G | A | G | A | T | C | C | A | C | C |
| SpCas9-pBE | WT |  |  | 53.3 |  |  |  |  |  |  |  |  |  |  |  |  |  |  |  |  |  |
|  | modified |  |  | 53.3 |  |  |  |  |  |  |  |  |  |  |  |  |  |  |  |  |  |
| eSpCas9(1.1)-pBE | WT |  |  | 6.7 |  |  |  |  |  |  |  |  |  |  |  |  |  |  |  |  |  |
|  | modified |  |  | 93.3 |  |  |  |  |  |  |  |  |  |  |  |  |  |  |  |  |  |
| SpCas9-HF2-pBE | WT |  |  | 15 |  |  |  |  |  |  |  |  |  |  |  |  |  |  |  |  |  |
|  | modified |  |  | 53.3 |  |  |  |  |  |  |  |  |  |  |  |  |  |  |  |  |  |
| HypaCas9-pBE | WT |  |  | 53.3 |  |  |  |  |  |  |  |  |  |  |  |  |  |  |  |  |  |
|  | modified |  |  | 33.3 |  |  |  |  |  |  |  |  |  |  |  |  |  |  |  |  |  |

|  | **NRT1.1B** | 1 | 2 | 3 | 4 | 5 | 6 | 7 | 8 | 9 | 10 | 11 | 12 | 13 | 14 | 15 | 16 | 17 | 18 | 19 | 20 |
| --- | --- | --- | --- | --- | --- | --- | --- | --- | --- | --- | --- | --- | --- | --- | --- | --- | --- | --- | --- | --- | --- |
| **Base Editor** | **sgRNA** | C | G | G | C | G | A | C | G | G | C | G | A | G | C | A | A | G | T | G | G |
| SpCas9-pBE | WT |  |  |  | 100 |  |  |  |  |  |  |  |  |  |  |  |  |  |  |  |  |
|  | modified | 6.7 |  |  | 60 |  |  |  |  |  |  |  |  |  |  |  |  |  |  |  |  |
| eSpCas9(1.1)-pBE | WT |  |  |  | 6.7 |  |  |  |  |  |  |  |  |  |  |  |  |  |  |  |  |
|  | modified |  |  |  | 86.7 |  |  |  |  |  |  |  |  |  |  |  |  |  |  |  |  |
| SpCas9-HF2-pBE | WT |  |  |  | 65 |  |  |  |  |  |  |  |  |  |  |  |  |  |  |  |  |
|  | modified |  |  |  | 86.7 |  |  |  |  |  |  |  |  |  |  |  |  |  |  |  |  |
| HypaCas9-pBE | WT |  |  |  | 86.7 |  |  |  |  |  |  |  |  |  |  |  |  |  |  |  |  |
|  | modified |  |  |  | 93.3 |  |  |  |  |  |  |  |  |  |  |  |  |  |  |  |  |

**Table S4.** The frequency of random mutation occurred during precise base editing on OsCDC48, OsALS and OsNRT1.1B sites in all SpCas9 base editors.

| **Base Editor** | **sgRNA** | **CDC48** | **ALS** | **NRT1.1B** |
| --- | --- | --- | --- | --- |
| SpCas9-pBE | WT | 0 | 0 | 62.5 |
| eSpCas9(1.1)-pBE | WT | 0 | 0 | 0 |
| SpCas9-HF2-pBE | WT | 0.0 | 0.0 | 42.9 |
| HypaCas9-pBE | WT | 0.0 | 0.0 | 53.8 |
| SpCas9-pBE | modified | 9.1 | 0.0 | 55.6 |
| eSpCas9(1.1)-pBE | modified | 9.1 | 0.0 | 92.3 |
| SpCas9-HF2-pBE | modified | 0.0 | 12.5 | 15.4 |
| HypaCas9-pBE | modified | 0.0 | 33.3 | 35.7 |

**Table S5.** Potential off-target sites in rice genomics for base editing targets

| Targets | Chromosome | Position | Guide-PAM Sequences | Mismatches Number |
| --- | --- | --- | --- | --- |
| CDC48 | 3 | 2831809 | GACCAGCCAGCGTCTGGCGCCGG | 0 |
| CDC48-OT1 | 12 | 5117923 | GACCAGCCgGCGTgTGGtGCAGG | 2 |
| CDC48-OT2 | 12 | 8239582 | GACCAGCCgGCGTgTGGtGCAGG | 2 |
| CDC48-OT4 | 4 | 20611300 | GACCAagCAGCGgCTGGCGCCGG | 3 |
| ALS | 2 | 18236404 | CGCGTCCATGGAGATCCACCAGG | 0 |
| ALS-OT1 | 4 | 19169517 | CGCGTCaATGGAGATCCACCAGG | 1 |
| ALS-OT2 | 4 | 19143688 | CGCGTCgATGGAGATCCACCAGG | 1 |
| ALS-OT3 | 10 | 4629927 | CGCGTCCAaGGAGtTCCAgCAGG | 3 |
| NRT1.1B | 10 | 21759074 | CGGCGACGGCGAGCAAGTGGAGG | 0 |
| NRT1.1B-OT2 | 5 | 2259825 | CGGCGACGGCGAGggAGTGGTGG | 2 |
| NRT1.1B-OT3 | 10 | 18999111 | CGGCGACGGCGAGCAtcTGGTGG | 2 |
| NRT1.1B-OT4 | 11 | 3734617 | CGGCGACGGCGAGCtcGTGGTGG | 2 |

**Table S6.** Potential off-target sites in rice genomics for random mutation targets

| Targets | Chromosome | Position | Guide-PAM Sequences | Mismatches Number |
| --- | --- | --- | --- | --- |
| GW8-T1 | 8 | 26505516 | GCAAGATGTTCTCCGATGGTGGG | 0 |
| GW8-T1-OT1 | 8 | 4442211 | GaAAcATGTTCTCaGATGGTGGG | 3 |
| GW8-T1-OT2 | 1 | 764468 | GCAAGgcGTTCTCCcATGGTGGG | 3 |
| GW8-T1-OT3 | 3 | 34007619 | GCAtGgTGcTCaCCGATGGTGGG | 4 |
| GW8-T1-OT4 | 6 | 4482235 | cCAAGcTtTTCaCCGATGGTCGG | 4 |
| GW8-T2 | 8 | 26505568 | TCTCTCTCTTCTGTCAGCTCCGG | 0 |
| GW8-T2-OT1 | 2 | 14338720 | TCTCTCTCTTCTGTCAGtTCCGG | 1 |
| GW8-T2-OT2 | 7 | 9541891 | TCTCTCTCTgCTGTCAaCTCCGG | 2 |
| GW8-T2-OT3 | 9 | 19647852 | TCTCTCTCTTCTGTCAtCcCCGG | 2 |
| GW8-T2-OT4 | 11 | 3518166 | TtTCTCTCTcCTcTCAGCTCTGG | 3 |

**Table S7.** Primers used in this study.

| Primer name | Primer sequence (5’-3’) | Purpose |
| --- | --- | --- |
| prOsUbq-F | tgctaagcttacaaattcgggtcaaggcgg | Amplifying OsUbq promoter |
| PrOsUbq-R | ccattacgtactgcaagaaataatcaccaa |  |
| prZmUbi1-F | tccgcctaggctgcagtgcagcgtgacc | Amplifying ZmUbi1 promoter |
| prZmUbi1-R | tcatgagctcctgcagaagtaacaccaaacaac |  |
| Hpt-F | gcaggagctcatgaaaaagcctgaactcaccgcg | Amplifying Hygromycin and terminator |
| T35S-R1 | gcgtcctgcaggctgaattaacgccgaattaa |  |
| SpCas9n-F | ccgaggtctcggtctatggactacaaggaccacgacg | Amplifying SpCas9 |
| SpCas9n-R | ccgaggtctcggctctttcttcttcttagcctgtcctgcc |  |
| UGI-F | ccgaggtctcggagctctccggcggcagcacgaacc | Amplifying UGI and terminator |
| T35S-R2 | cgcccctaggccactggattttggttttaggaatta |  |
| eSpCas9-F1 | tccttgcagacgattcaatcgataataaggtgttg | eSpCas9n mutation |
| eSpCas9-R1 | actccaatgcagggtatttcttaataagggcggtc |  |
| eSpCas9-F2 | gaaataccctgcattggagtctgaattcgtttacg |  |
| eSpCas9-R2 | gagaggtgcctttctaatttcgccatttgccag |  |
| eSpCas9-F3 | aaattagaaaggcacctctcatagagactaacggt |  |
| eSpCas9-R3 | gattgaatcgtctgcaaggaaagattgcgggaca |  |
| SpCas9-HF2n-F1 | ctgccttcgataagaacctgccgaacgagaaagttct | SpCas9-HF2n mutation |
| SpCas9-HF2n-R1 | gctttctagacagagcgccccatccagtgtaccttc |  |
| SpCas9-HF2n-F2 | gggcgctctgtctagaaagctcatcaacggaatacgt |  |
| SpCas9-HF2n-R2 | tcatgaatcagcgccataaaatttctgttggcgaatc |  |
| SpCas9-HF2n-F3 | tttatggcgctgattcatgacgatagtctcaccttc |  |
| SpCas9-HF2n-R3 | gacaatgtggtccacatcatagtcggacaaacggttg |  |
| SpCas9-HF2n-F4 | atgatgtggaccacattgtcccgcaatctttccttaag |  |
| SpCas9-HF2n-R4 | ttcgtgatcgctctagtctcaacgagctgacgctta |  |
| SpCas9-HF2n-F5 | gagactagagcgatcacgaagcatgtcgcgcagatt |  |
| SpCas9-HF2n-R5 | caggttcttatcgaaggcagtcatcctctcaatgaat |  |
| HypaCas9-F1 | gcttttgcggcactgattgctgacgatagtctcaccttca | HypaCas9n mutation |
| HypaCas9-R1 | gtccggtaatagattggtggatgagggtggcatccagcac |  |
| HypaCas9-F2 | ccaccaatctattaccggactctacgagactagaatc |  |
| HypaCas9-R2 | agcaatcagtgccgcaaaagctctgttggcgaatccgtccg |  |
| BE-T1-F | Cactggtctcatgcacgcgtccatggagatccaccgttttagagctagaaatagcaag | SgRNA-wt BE construction |
| BE-T1-R | Cactggtctcaacgctggctggtctgcaccagccgggaatcg |  |
| BE-T2-F | cactggtctcagcgtctggcgcgttttagagctagaaatagcaag |  |
| BE-T2-R | cactggtctcaaaacccacttgctcgccgtcgccgtgcaccagccgggaatcg |  |
| BE-T1M-F | cactggtctcatgcacgcgtccatggagatccaccgtttcagagctatgctggaaac | sgRNA-modified BE construction |
| BE-T1M-R | cactggtctcaacgctggctggtctgcaccagccgggaatcg |  |
| BE-T2M-F | cactggtctcagcgtctggcgcgtttcagagctatgctggaaac |  |
| BE-T2M-R | cactggtctcaaaacccacttgctcgccgtcgccgtgcaccagccgggaatcg |  |
| KO-T1-F | cactggtctcatgcagcacaggccacatccttctcgttttagagctagaaatagcaag | SgRNA-wt KO construction |
| KO-T1-R | cactggtctcagagaacatcttgctgcaccagccgggaatcg |  |
| KO-T2-F | cactggtctcatctccgatggtgttttagagctagaaatagcaag |  |
| KO-T2-R | cactggtctcaaaacgagctgacagaagagagagatgcaccagccgggaatc |  |
| KO-T1M-F | cactggtctcatgcagcacaggccacatccttctcgtttcagagctatgctggaaac | sgRNA-modified KO construction |
| KO-T1M-R | cactggtctcagagaacatcttgctgcaccagccgggaatcg |  |
| KO-T2M-F | cactggtctcatctccgatggtgtttcagagctatgctggaaac |  |
| KO-T2M-R | cactggtctcaaaacgagctgacagaagagagagatgcaccagccgggaatc |  |
| NRT-MOT1-F1 | agtcagggtctcttgcatagcgacggcgagcaagtgg | Off-target constructions of NRT1.1B |
| NRT-MOT1-R1 | agtcagggtctctaaacccacttgctcgccgtcgcta |  |
| NRT-MOT1-F2 | agtcagggtctcttgcacgatgacggcgagcaagtgg |  |
| NRT-MOT1-R2 | agtcagggtctctaaacccacttgctcgccgtcatcg |  |
| NRT-MOT1-F3 | agtcagggtctcttgcacggcagcggcgagcaagtgg |  |
| NRT-MOT1-R3 | agtcagggtctctaaacccacttgctcgccgctgccg |  |
| NRT-MOT1-F4 | agtcagggtctcttgcacggcgatagcgagcaagtgg |  |
| NRT-MOT1-R4 | agtcagggtctctaaacccacttgctcgctatcgccg |  |
| NRT-MOT1-F5 | agtcagggtctcttgcacggcgacgatgagcaagtgg |  |
| NRT-MOT1-R5 | agtcagggtctctaaacccacttgctcatcgtcgccg |  |
| NRT-MOT1-F6 | agtcagggtctcttgcacggcgacggcaggcaagtgg |  |
| NRT-MOT1-R6 | agtcagggtctctaaacccacttgcctgccgtcgccg |  |
| NRT-MOT1-F7 | agtcagggtctcttgcacggcgacggcgaataagtgg |  |
| NRT-MOT1-R7 | agtcagggtctctaaacccacttattcgccgtcgccg |  |
| NRT-MOT1-F8 | agtcagggtctcttgcacggcgacggcgagcgggtgg |  |
| NRT-MOT1-R8 | agtcagggtctctaaacccacccgctcgccgtcgccg |  |
| NRT-MOT1-F9 | agtcagggtctcttgcacggcgacggcgagcaaacgg |  |
| NRT-MOT1-R9 | agtcagggtctctaaacccgtttgctcgccgtcgccg |  |
| NRT-MOT1-F10 | agtcagggtctcttgcacggcgacggcgagcaagtaa |  |
| NRT-MOT1-R10 | agtcagggtctctaaacttacttgctcgccgtcgccg |  |
| CDC48-F | acatcgagatggagaagcgg | Genomic amplify  (on target) |
| CDC48-R | ccatgctccaatcgatgaatac |  |
| ALS-F | taagaaccaccagcgacacc |  |
| ALS-R | ggtaattgtgcttggtgatggag |  |
| NRT1.1B-F | ttacgaactttataactttgtcgg |  |
| NRT1.1B-R | atggaggcgatgaggaagac |  |
| Ghd7-F | tgatcgagctcaagtgacctc |  |
| Ghd7-R | aagaactggaactcgtgcacc |  |
| GW8-F | catttcgttggctccacctc |  |
| GW8-R | ccagagatgagaggctgcg |  |

**List of legends for supplementary tables**

**Table S1.** Comparation of base editing efficiency in our study with reported results.

**Table S2.** Base editing preference of SpCas9-rBE and SpCas9-pBE in rice.

**Table S3.** Precise base editing preferences and frequencies in all SpCas9 base editors on OsCDC48, OsALS and OsNRT1.1B sites.

**Table S4.** The frequency of random mutation occurred during precise base editing on OsCDC48, OsALS and OsNRT1.1B sites in all SpCas9 base editors.

**Table S5.** Potential off-target sites in rice genomics for base editing targets.

**Table S6.** Potential off-target sites in rice genomics for random mutation targets.

**Table S7.** Primers used in this study.
